# Association of histologic and clinical activity with major adverse cardiovascular events in patients with inflammatory bowel disease: A cohort study

**DOI:** 10.1111/joim.70035

**Published:** 2025-10-23

**Authors:** Jiangwei Sun, Karl Mårild, Johan Sundström, David Bergman, SWIBREG Study Group, Fahim Ebrahimi, Jonas Halfvarson, Ola Olén, Jonas F. Ludvigsson

**Affiliations:** ^1^ Department of Medical Epidemiology and Biostatistics Karolinska Institutet Stockholm Sweden; ^2^ Department of Pediatrics, Institute of Clinical Sciences Sahlgrenska Academy Gothenburg Sweden; ^3^ Department of Pediatrics Queen Silvia Children's Hospital Gothenburg Sweden; ^4^ Department of Medical Sciences Uppsala University Uppsala Sweden; ^5^ The George Institute for Global Health University of New South Wales Sydney Australia; ^6^ Department of Gastroenterology and Hepatology University Digestive Health Care Center Basel—Clarunis Basel Switzerland; ^7^ Department of Gastroenterology, Faculty of Medicine and Health Örebro University Örebro Sweden; ^8^ Division of Clinical Epidemiology, Department of Medicine, Solna Karolinska Institutet Stockholm Sweden; ^9^ Sachs’ Children and Youth Hospital Stockholm South General Hospital Stockholm Sweden; ^10^ Department of Pediatrics Örebro University Hospital Örebro Sweden; ^11^ Division of Digestive and Liver Disease, Department of Medicine Columbia University Medical Center New York New York USA

**Keywords:** clinical activity, cohort, histologic remission, inflammatory bowel disease, MACE

## Abstract

**Objectives:**

Inflammatory bowel disease (IBD) is a chronic disorder linked to cardiovascular disease (CVD). However, the impact of histologic and clinical activity on this association remains unclear.

**Methods:**

We conducted a nationwide cohort study in Sweden involving 59,168 IBD patients diagnosed in 1969–2017 with histologic evaluation and 91,800 patients diagnosed in 1969–2020 with assessment of clinical activity in 2006–2021. The primary outcome was incident major adverse cardiovascular events (MACE), a composite outcome encompassing ischemic heart disease, stroke, and heart failure. Cox proportional hazards model estimated adjusted hazard ratios (aHRs) of MACE and its subcomponents.

**Results:**

We found an increased MACE risk following histologic inflammation (*n* = 868, incidence rate [IR]: 86.3/10,000 person‐years) compared to remission (*n* = 558, IR = 71.3) (aHR = 1.16 [1.04–1.30]). This excess risk was evident in Crohn's disease (aHR = 1.30 [1.03–1.64]) and ulcerative colitis (aHR = 1.13 [1.01–1.27]). Histologic inflammation was associated with an increased risk of ischemic heart disease, myocardial infarction, ischemic stroke, and heart failure, but not with hemorrhagic stroke. Compared to clinically quiescent IBD, active IBD was associated with an increased MACE risk (IR: 131.4 vs. 93.7; aHR = 1.54 [1.46–1.63]) and all MACE subcomponents. In patients with clinically quiescent IBD, histologic inflammation remained linked to myocardial infarction (aHR = 1.29 [1.06–1.58]) and heart failure (aHR = 1.19 [1.00–1.43]).

**Conclusion:**

Both histologic and clinical activities of IBD were associated with an increased MACE risk, suggesting that improved disease control may reduce MACE risk in IBD.

## Graphical abstract

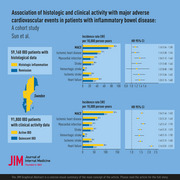

## Introduction

Inflammatory bowel disease (IBD), encompassing Crohn's disease (CD), ulcerative colitis (UC), and IBD‐unclassified (IBD‐U), is a chronic systemic disease that primarily affects the gastrointestinal (GI) tract but also has extraintestinal manifestations and comorbidities [1, 2, 3]. Among these, cardiovascular disease (CVD) is a leading cause of disability and death [4].

IBD has been linked to increased major adverse cardiovascular events [5, 6] (MACE, including ischemic heart disease [7], stroke [8], and heart failure [9]). However, considering that the clinical course of IBD follows a relapsing–remitting pattern [10], it is still unclear whether MACE risk varies across different disease activity periods. Although studies have suggested a heightened risk of CVD during intense disease phases (e.g., flares) [11, 12, 13, 14, 15, 16, 17], most of these studies have suffered from limitations (e.g., small sample sizes or short follow‐ups). Moreover, because histologic activity may persist even in patients without clinical or endoscopic signs of disease activity, histologic remission is increasingly recognized as a therapeutic endpoint in IBD (particularly in UC [2]), and accumulating evidence suggests that achieving histologic remission is associated with improved clinical outcomes [18, 19, 20, 21, 22]. However, the association between histologic activity and the risk of MACE remains uncertain. Clinically, it is important to determine whether histologic inflammation is associated with MACE even in clinically quiescent IBD, given the established role of inflammation on CVD development (e.g., atherosclerosis) and progression from endothelial dysfunction to clinical syndromes [23].

Therefore, in this nationwide, population‐based cohort, we sought to investigate the role of histologic inflammation (vs. histologic remission) and clinically active IBD (vs. quiescent IBD) on the risk of MACE.

## Methods

### Data source and study population

Multiple Swedish national registers were used in this study (eMethods). IBD patients were identified as those having either ≥2 International Classification of Disease (ICD) codes in the National Patient Register (NPR) [24] or ≥1 ICD code plus ≥1 IBD‐indicative biopsy in ESPRESSO (Epidemiology Strengthened by histoPathology Reports in Sweden [25], Table S1). This definition has been validated with a positive predictive value (PPV) of 93%–95% [26, 27, 28]. To avoid immortal time bias, the IBD diagnosis date was set when both criteria were met. We classified IBD into three subtypes: CD, UC, and IBD‐U. At diagnosis, IBD phenotypes were classified using the Montreal classification criteria (already validated in Sweden [29]), including CD location and perianal disease modifier, UC extent, presence of primary sclerosing cholangitis (PSC), and other extraintestinal manifestations (Table S2).

We studied the associations of histologic and clinical activity with MACE by identifying two mutually nonexclusive IBD groups based on the following inclusion criteria: the first group comprised 59,168 patients diagnosed in 1969–2017 with available information on histology; the second group included 91,800 patients diagnosed in 1969–2020 and residing in Sweden after 2006, with information on clinical activity between 1 January 2006 and 31 December 2021.

### Exposures

Aligned with expert consensus [30, 31] and prior studies [21, 22], histologic inflammation (primary exposure) was defined as having ≥1 SNOMED (the Swedish version of the Systematized Nomenclature of Medicine) code for ileocolorectal inflammation or ulceration/erosion (Table S3). Histologic remission was defined through ileocolorectal histopathology codes M00100/M00110 (normal mucosa) [25] and the absence of SNOMED codes indicative of inflammation, ulceration, or erosion. While recognizing the diverse trajectories of disease in IBD patients [10], we hypothesized that histologic inflammation would persist for 12 months after biopsy [21], with an additional 12 months if another inflammation biopsy was documented within the initial 12 months.

Consistent with our previous publications [21, 22], we identified periods of clinically active IBD (secondary exposure) through the following events: (a) IBD‐related surgery (Table S4), (b) IBD‐related hospitalization, (c) ≥1 corticosteroid dispensation (either oral or topical therapy), or (d) initiation of immunomodulators or targeted therapies. In line with earlier research [21, 32], a clinically active period would persist for up to 3 months following any identified clinical events. The active period would extend for an additional 3 months if another event occurred within 3 months of the prior event [21, 22]. Periods without signs of clinical activity were defined as clinically quiescent. We identified IBD medication from the NPR [24], the Prescribed Drug Register [PDR] [33], and the Swedish Quality Register for IBD (SWIBREG) [34] (Table S5). Because the PDR has been available only since July 2005, we limited our clinical activity study to January 2006 and onwards to capture at least 6 months of drug data.

Of note, both histologic and clinical activity were treated as a time‐varying exposure (i.e., exposure status was not necessarily constant through follow‐up).

### Outcomes and follow‐up

Our primary outcome was newly diagnosed MACE, a composite outcome encompassing diagnoses of any secondary outcomes (i.e., ischemic heart disease [myocardial infarction], stroke [hemorrhagic stroke and ischemic stroke], and heart failure) (Table S6). Individuals with each of those secondary outcomes were identified from the NPR, considering both primary and secondary diagnoses, with a PPV of 98%–100% for myocardial infarction [24], 94% for stroke [35], and 82% for heart failure [36]. If an individual was diagnosed with multiple secondary outcomes, this individual contributed to each outcome with the respective diagnosis date.

The index date was defined as the date of documented histologic inflammation or remission and the date of clinically active or quiescent IBD. Individuals with a diagnosis of MACE before the index date were excluded from the analysis. Follow‐up started at the index date and ended at diagnosis of incident MACE, change in exposure status, emigration, 2 years after the index date, or end of follow‐up on 31 December 2021, whichever occurred first. We assumed that the effect of histologic inflammation would have an impact on MACE risk within 2 years and therefore chose incident MACE within 2 years after the index date as our main analysis. Additionally, to address the potential arbitrariness of assuming the duration of the histologic inflammation effect, we conducted a sensitivity analysis to assess the risk of incident MACE within a 5‐year period after the index date.

### Covariates

We included age at IBD diagnosis, sex, and calendar year at index date in model 1. In Model 2, we additionally adjusted for county of residence, educational attainment, country of birth, number of health care visits, and history of CVD‐related comorbidities (including hypertension, diabetes, obesity, dyslipidemia, chronic kidney disease, and chronic obstructive pulmonary disease [COPD]) (see eMethods for details and Table S6 for their definitions). All covariates, except age at IBD diagnosis, sex, and country of birth, were updated at the index date (i.e., time‐varying covariates).

### Statistical analyses

We applied Cox proportional hazards model to estimate hazard ratios (HRs) with 95% confidence intervals (CIs) for primary and secondary outcomes after histologic inflammation (vs. histologic remission) and clinically active IBD (vs. quiescent IBD), using age at the index date as the underlying time scale. An evaluation of proportional hazard assumption was conducted using Schoenfeld residuals, revealing no violations. The analyses were run first for IBD and then for its subtypes (i.e., CD, UC, and IBD‐U). Robust standard errors accounted for correlations between periods from the same individual. We used Poisson regression to estimate incidence rates (IRs) of MACE.

### Subgroup and sensitivity analysis

We estimated HRs for MACE by sex, age at IBD diagnosis, age at index date, calendar period at IBD diagnosis, IBD duration (0, >0–5, and >5 years), the number of health care visits, and history of CVD‐related comorbidities before the index date. To investigate the potential influence of introducing modern IBD therapy, we stratified the analysis by calendar period. We additionally calculated phenotype‐specific HRs by CD location, UC extent, and with PSC or other extraintestinal manifestations.

To examine the impact of the defined duration of histologic inflammation and clinically active IBD, we repeated the analyses, modifying the presumed duration of histologic inflammation from 1 year to 6 months and the clinically active IBD duration from 3 to 6 months. Moreover, to estimate the possible incremental effect of residual histologic inflammation on MACE risk in patients with clinically quiescent IBD, we restricted data to 2006 and 2017 and estimated 2‐year MACE risk for histologic inflammation (vs. remission) during quiescent IBD periods. Furthermore, to explore the association of histologic inflammation and MACE beyond the period near IBD diagnosis, we discarded the first 3 months or 1 year after IBD diagnosis from the analysis.

Data analyses were performed using SAS version 9.4 and R version 3.6.0. A two‐sided *p *≤ 0.05 was considered statistically significant.

## Results

### Histologic inflammation versus remission

Some 59,168 IBD patients (median age at diagnosis: 36.2 years) were enrolled, including 17,093 with CD, 38,877 with UC, and 3198 with IBD‐U (Table S7). The cohort included 48.1% female, 11.4% with childhood‐onset IBD, and 54% diagnosed since 2002 (Table S7). Some 51,717 contributed to 99,123 inflammation periods (median age at index date: 42.7 years; median [interquartile range, IQR] duration of inflammation: 1.00 [1.00–1.00] year), and 26,966 patients contributed to 46,049 remission periods (45.6 years; median [IQR] duration of remission: 2.00 [1.64–2.00] years) (Table 1). More remission periods were recorded after 2001 (Table 1). Approximately 30% of histologic inflammation periods were within the first 3 months after IBD diagnosis (Fig. S1).

**Table 1:** Characteristics of inflammatory bowel disease (IBD) patients during periods of histologic inflammation and histologic remission in 1969–2017.

	Histologic remission	Histologic inflammation	Histologic inflammation in subtypes of IBD		
			CD	UC	IBD‐U
No. of periods	46,049	99,123	25,488	68,133	5502
No. of patients	26,966	51,717	14,597	34,253	2867
No. of patients by no. of periods					
1	16,685 (61.9)	28,962 (56.0)	8661 (59.3)	18,605 (54.3)	1696 (59.2)
2	5782 (21.4)	11,403 (22.1)	3351 (23.0)	7487 (21.9)	565 (19.7)
3	2398 (8.9)	5330 (10.3)	1369 (9.4)	3715 (10.9)	246 (8.6)
≥4	2101 (7.8)	6022 (11.6)	1216 (8.3)	4446 (13.0)	360 (12.6)
Age, years^a^					
Mean ± SD	45.2 ± 16.4	43.4 ± 17.2	40.6 ± 17.3	44.4 ± 17.0	44.4 ± 17.6
Median (IQR)	45.6 (32.9–57.8)	42.7 (30.0–56.3)	39.2 (26.6–53.8)	43.8 (31.3–57.0)	44.6 (30.4–57.9)
<18	2319 (5.0)	6013 (6.1)	2300 (9.0)	3346 (4.9)	367 (6.7)
18–<40	15,348 (33.3)	38,148 (38.5)	10,838 (42.5)	25,394 (37.3)	1916 (34.8)
40–<60	18,848 (40.9)	35,990 (36.3)	8275 (32.5)	25,662 (37.7)	2053 (37.3)
≥60	9534 (20.7)	18,972 (19.1)	4075 (16.0)	13,731 (20.2)	1166 (21.2)
Calendar period^a^					
1969–1989	1234 (2.7)	6231 (6.3)	1707 (6.7)	3953 (5.8)	571 (10.4)
1990–2001	7757 (16.9)	28,803 (29.1)	7219 (28.3)	20,098 (29.5)	1486 (27.0)
2002–2009	15,079 (32.8)	31,284 (31.6)	7962 (31.2)	22,023 (32.3)	1299 (23.6)
2010‐2017	21,979 (47.7)	32,805 (33.1)	8600 (33.7)	22,059 (32.4)	2146 (39.0)
Educational attainment^a^					
0–9 years	8623 (18.7)	20,440 (20.6)	5436 (21.3)	13,790 (20.2)	1214 (22.1)
10–12 years	20,020 (43.5)	42,187 (42.6)	10,937 (42.9)	29,041 (42.6)	2209 (40.2)
≥13 years	14,641 (31.8)	25,965 (26.2)	5857 (23.0)	18,849 (27.7)	1259 (22.9)
Missing	2765 (6.0)	10,531 (10.6)	3258 (12.8)	6453 (9.5)	820 (14.9)
IBD duration, years^a^					
Mean ± SD	9.2 ± 8.5	6.6 ± 7.9	6.6 ± 8.3	6.5 ± 7.7	7.8 ± 9.4
Median (IQR)	7.3 (2.1–13.7)	3.5 (0.0–10.7)	3.1 (0.0–10.6)	3.7 (0.0–10.6)	3.7 (0.0–13.2)
0	1932 (4.2)	27,172 (27.4)	7253 (28.5)	18,503 (27.2)	1416 (25.7)
>0–5	16,276 (35.3)	28,192 (28.4)	7365 (28.9)	19,276 (28.3)	1551 (28.2)
>5	27,841 (60.5)	43,759 (44.2)	10,870 (42.7)	30,354 (44.6)	2535 (46.1)
Number of health care visits^b^					
0	15,561 (33.8)	45,149 (45.6)	10,889 (42.7)	31,785 (46.7)	2475 (45.0)
1	9832 (21.4)	19,446 (19.6)	4677 (18.4)	13,701 (20.1)	1068 (19.4)
2–3	9655 (21.0)	17,502 (17.7)	4581 (18.0)	11,972 (17.6)	949 (17.3)
≥4	11,001 (23.9)	17,026 (17.2)	5341 (21.0)	10,675 (15.7)	1010 (18.4)
Disease history^a^					
Hypertension	3275 (7.1)	5463 (5.5)	1336 (5.2)	3702 (5.4)	425 (7.7)
Diabetes	1909 (4.2)	3494 (3.5)	693 (2.7)	2520 (3.7)	281 (5.1)
Obesity	745 (1.6)	1218 (1.2)	376 (1.5)	760 (1.1)	82 (1.5)
Dyslipidemia	650 (1.4)	802 (0.8)	172 (0.7)	579 (0.9)	51 (0.9)
Chronic kidney diseases	445 (1.0)	801 (0.8)	229 (0.9)	512 (0.8)	60 (1.1)
COPD	488 (1.1)	1136 (1.2)	297 (1.2)	753 (1.1)	86 (1.6)

Abbreviations: CD, Crohn's disease; COPD, chronic obstructive pulmonary disease; IBD(‐U), inflammatory bowel disease (unclassified); IQR, interquartile range; SD, standard deviation; UC, ulcerative colitis.

^a^ Measured at index date. Index date was defined as date of histologic inflammation or remission.

^b^ Measured between 2 years and 6 months before the index date.

**Fig. 1:**
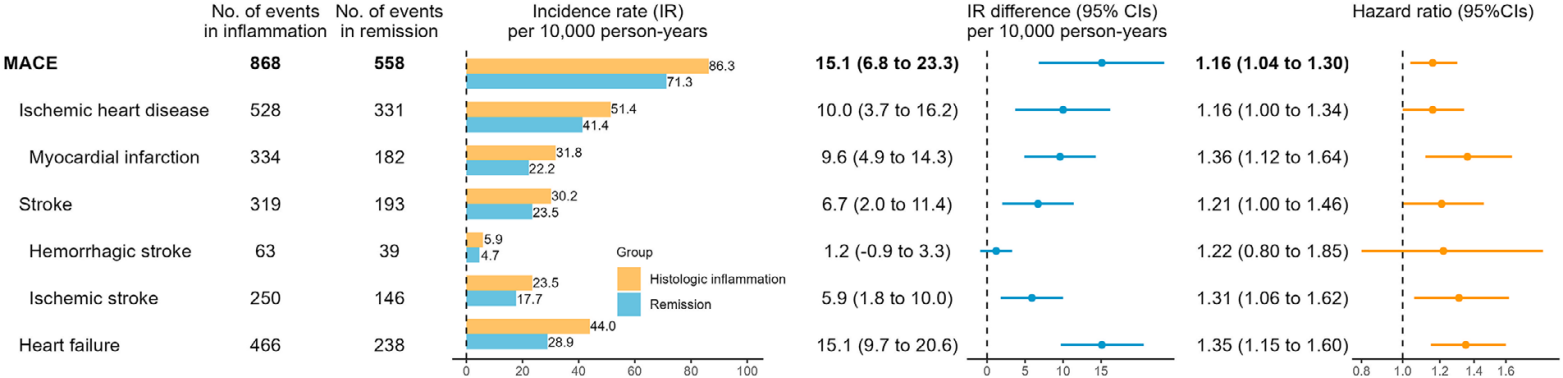
Incidence rates (IRs) and hazard ratios for the 2‐year major adverse cardiovascular events (MACE) for histologic inflammation (vs. remission), with 95% confidence intervals (CIs). The hazard ratios were estimated from the Cox proportional hazards model based on Model 2.

We identified 868 incident MACE after histologic inflammation (IR: 86.3/10,000 person‐years) and 558 after remission (IR = 71.3), with an IR difference of 15.1 (95% CI: 6.8–23.3) (Fig. 1 and Table S8). These rates correspond to one additional MACE per 331 IBD patients over 2 years after histologic inflammation, with an adjusted HR (aHR) of 1.16 (1.04–1.30). The association was significant for all secondary outcomes including ischemic heart disease (aHR = 1.16 [1.04–1.30]), myocardial infarction (aHR = 1.36 [1.12–1.64]), ischemic stroke (aHR = 1.31 [1.06–1.62]), and heart failure (aHR = 1.35 [1.15–1.60]), except hemorrhagic stroke (aHR = 1.22 [0.80–1.85]).

The increased risk of MACE after histologic inflammation was also observed in CD (aHR = 1.30 [1.03–1.64]) and UC (aHR = 1.13 [1.01–1.27]) but was not significant in IBD‐U (aHR = 1.37 [0.85–2.21]) (Table S9). For secondary outcomes, histologic inflammation was associated with an increased risk of heart failure in CD, myocardial infarction and heart failure in UC, and ischemic stroke in IBD‐U.

As expected, the IR for MACE increased with age, the number of health care visits, and CVD‐related comorbidities (Table S10). Compared to remission, histologic inflammation was associated with a higher relative risk of MACE in females, in IBD patients diagnosed ≥60 years old, and in those diagnosed with IBD after 2002 (Table S10). An increased risk for MACE after histologic inflammation was also observed in patients with PSC (aHR = 7.22 [1.02–51.04]), other extraintestinal manifestations (aHR = 2.11 [1.08–4.13]), and colonic CD (aHR = 1.96 [1.08–3.56]) (Table S11), although data on IBD phenotypes were only available for a subset of the population and number of events was small (Table S7).

In sensitivity analyses, comparable results for MACE were observed when extending the follow‐up to 5 years (aHR = 1.17 [1.07–1.28], Table S12) and assuming a histologic inflammation duration of 6 months (aHR = 1.28 [1.13–1.45], Table S13). Histologic inflammation (vs. remission) was also linked to an increased risk of myocardial infarction (aHR = 1.29 [1.06–1.58]) and heart failure (aHR = 1.19 [1.00–1.43]) in patients with clinically quiescent IBD (Table S14). After excluding the first 3 months or 1 year after IBD diagnosis from the analysis, the IR of MACE decreased, particularly in histologic inflammation (Table S15). Although the risk of overall MACE was no longer increased, we still observed significantly increased relative risks between histologic inflammation and myocardial infarction and heart failure (Table S15).

### Clinically active versus quiescent IBD

This analysis comprised 91,800 IBD patients diagnosed in 1969–2020: 30,729 with CD, 54,015 with UC, and 7056 with IBD‐U (Table S16). They contributed to 274,568 periods of clinically active IBD (median age at index date: 48.0 years; median [IQR] duration of active IBD: 0.46 [0.27–0.98] years) and 277,939 quiescent periods (47.6 years; median [IQR] duration of quiescent IBD: 0.54 [0.13–2.00] years) (Table 2 and Fig. S2). Both groups had similar characteristics at the index date (Table 2).

**Table 2:** Characteristics of inflammatory bowel disease (IBD) patients during periods of clinically active and quiescent IBD in 2006–2020.

			Active IBD in subtypes of IBD		
	Quiescent IBD	Active IBD	CD	UC	IBD‐U
No. of periods	277,939	274,568	103,218	153,821	17,529
No. of patients	85,215	81,488	28,860	46,431	6197
No. of patients by no. of periods					
1	29,521 (34.6)	26,832 (32.9)	8941 (31.0)	15,331 (33.0)	2560 (41.3)
2	17,281 (20.3)	16,396 (20.1)	5642 (19.6)	9462 (20.4)	1292 (20.9)
3	11,422 (13.4)	11,083 (13.6)	3919 (13.6)	6393 (13.8)	771 (12.4)
≥4	26,991 (31.7)	27,177 (33.4)	10,358 (35.9)	15,245 (32.8)	1574 (25.4)
Age, years^a^					
Mean ± SD	47.9 ± 18.1	48.0 ± 18.4	47.2 ± 18.5	48.6 ± 18.1	47.7 ± 19.9
Median (IQR)	47.6 (33.1–62.2)	48.0 (33.0–62.6)	47.4 (31.9–61.9)	48.3 (34.0–62.9)	48.8 (30.2–64.1)
<18	9185 (3.3)	10,447 (3.8)	4721 (4.6)	4565 (3.0)	1161 (6.6)
18–<40	93,421 (33.6)	90,155 (32.8)	34,454 (33.4)	50,200 (32.6)	5501 (31.4)
40–<60	95,279 (34.3)	93,030 (33.9)	34,884 (33.8)	52,975 (34.4)	5171 (29.5)
≥60	80,054 (28.8)	80,936 (29.5)	29,159 (28.3)	46,081 (30.0)	5696 (32.5)
Calendar period^a^					
2006–2009	56,000 (20.2)	55,218 (20.1)	21,234 (20.6)	31,426 (20.4)	2558 (14.6)
2010–2020	221,939 (79.9)	219,350 (79.9)	81,984 (79.4)	122,395 (79.6)	14,971 (85.4)
Educational attainment^a^					
0–9 year	51,382 (18.5)	51,828 (18.9)	20,668 (20.0)	27,510 (17.9)	3650 (20.8)
10–12 year	127,322 (45.8)	124,728 (45.4)	47,527 (46.1)	69,726 (45.3)	7475 (42.6)
≥13 year	87,098 (31.3)	84,074 (30.6)	29,023 (28.1)	50,216 (32.7)	4835 (27.6)
Missing	12,137 (4.4)	13,938 (5.1)	6000 (5.8)	6369 (4.1)	1569 (9.0)
IBD duration, years^a^					
Mean ± SD	10.7 ± 10.2	11.0 ± 10.3	11.8 ± 11.1	10.6 ± 9.5	9.8 ± 11.9
Median (IQR)	8.0 (2.4–15.9)	8.3 (2.7–16.3)	8.8 (2.7–17.9)	8.4 (3.0–15.5)	4.7 (0.6–14.6)
0	25,612 (9.2)	30,270 (11.0)	11,986 (11.6)	14,875 (9.7)	3409 (19.5)
>0–5	77,678 (28.0)	67,699 (24.7)	23,956 (23.2)	38,179 (24.8)	5564 (31.7)
>5	174,649 (62.8)	176,599 (64.3)	67,276 (65.2)	100,767 (65.5)	8556 (48.8)
Number of health care visits^b^					
0	59,713 (21.5)	61,053 (22.2)	21,379 (20.7)	35,689 (23.2)	3985 (22.7)
1	52,993 (19.1)	52,927 (19.3)	19,492 (18.9)	30,583 (19.9)	2852 (16.3)
2–3	67,514 (24.3)	66,760 (24.3)	24,622 (23.9)	38,156 (24.8)	3982 (22.7)
≥4	97,719 (35.2)	93,828 (34.2)	37,725 (36.6)	49,393 (32.1)	6710 (38.3)
Disease history^a^					
Hypertension	37,441 (13.5)	36,063 (13.1)	13,243 (12.8)	19,836 (12.9)	2984 (17.0)
Diabetes	16,312 (5.9)	15,745 (5.7)	4939 (4.8)	9578 (6.2)	1228 (7.0)
Obesity	9654 (3.5)	9450 (3.4)	3628 (3.5)	5035 (3.3)	787 (4.5)
Dyslipidemia	6296 (2.3)	5975 (2.2)	1634 (1.6)	3866 (2.5)	475 (2.7)
Chronic kidney diseases	5855 (2.1)	5814 (2.1)	2351 (2.3)	2972 (1.9)	491 (2.8)
COPD	9238 (3.3)	9238 (3.4)	3747 (3.6)	4691 (3.1)	800 (4.6)

Abbreviations: CD, Crohn's disease; COPD, chronic obstructive pulmonary disease; IBD(‐U), inflammatory bowel disease (unclassified); IQR, interquartile range; SD, standard deviation; UC, ulcerative colitis.

^a^ Measured at index date. Index date was defined as date of clinically active or quiescent IBD.

^b^ Measured between 2 years and 6 months before the index date.

**Fig. 2:**
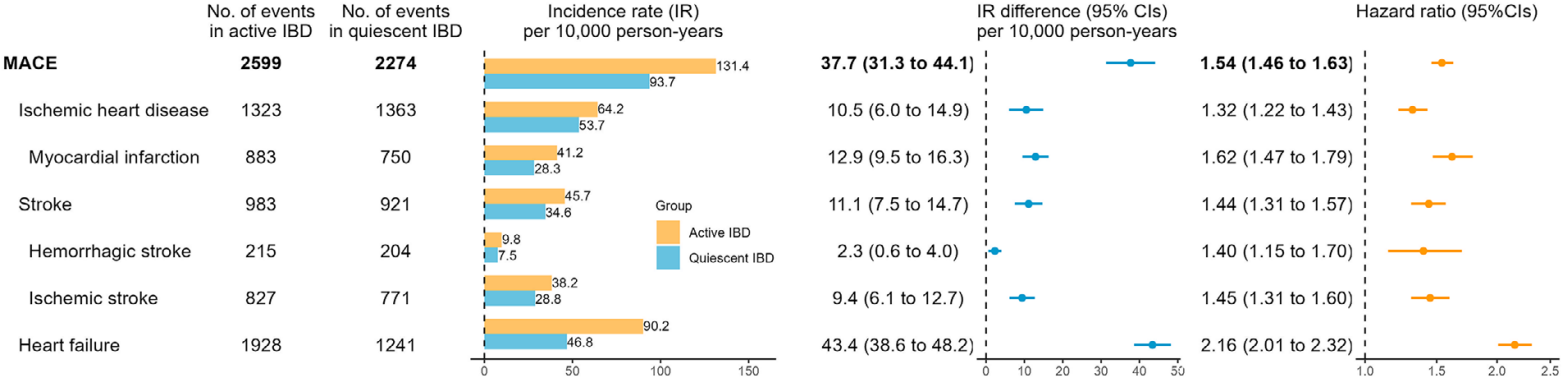
Incidence rates (IRs) and hazard ratios for the 2‐year major adverse cardiovascular events (MACE) for clinically active inflammatory bowel disease (IBD) (vs. quiescent IBD), with 95% confidence intervals (CIs). The hazard ratios were estimated from the Cox proportional hazards model based on Model 2.

We identified 2599 (IR = 131.4/10,000 person‐years) versus 2274 (IR = 93.7) incident MACE for active versus quiescent IBD, respectively, resulting in an IR difference of 37.7 (31.3–44.1). These rates correspond to one additional MACE per 133 IBD patients over 2 years after active IBD, with an aHR of 1.54 (1.46–1.63) (Fig. 2 and Table S17). aHRs for active IBD were also significantly increased for all secondary outcomes, for example, myocardial infarction (aHR = 1.62 [1.47–1.79]), ischemic stroke (aHR = 1.45 [1.31–1.60]), and heart failure (aHR = 2.16 [2.01–2.32]).

Active IBD was associated with excess MACE risks in CD (aHR = 1.50 [1.36–1.65]), UC (aHR = 1.53 [1.42–1.65]), and IBD‐U (aHR = 1.91 [1.56–2.35]) (Table S18). Additionally, elevated risks of secondary outcomes were noted across IBD subtypes.

Subgroup analyses revealed notable differences in the IR of MACE despite comparable increases in relative risk (Table S19). The IR for MACE increased with age and the number of health care visits while decreasing with educational attainment. Moreover, it was higher in individuals with CVD‐related comorbidities before the index date. The relative risk for MACE was higher in females and peaked shortly after IBD diagnosis (aHR = 1.94 [1.63–2.32]) but remained elevated for active IBD that occurred >5 years after diagnosis (aHR = 1.47 [1.38–1.58]) (Table S19). Stratified analysis by IBD phenotypes did not show significant differences in absolute or relative MACE risks (Table S20).

Robust associations in sensitivity analyses were observed when extending the follow‐up to 5 years (aHR = 1.57 [1.50–1.65], Table S21) and assuming a 6‐month duration of clinically active IBD (aHR = 1.65 [1.56–1.76], Table S22).

## Discussion

In this large nationwide cohort study, both histologic and clinical activity were linked to an increased risk of MACE, including myocardial infarction, ischemic stroke, and heart failure, suggesting that improved disease control may provide opportunities to reduce MACE risk in IBD.

A greater absolute risk of MACE development was observed in those with traditional CVD risk factors before the index date, including older age, low educational attainment, and comorbidities. For example, in every 10,000 person‐years, there were 337 incident MACE cases in those who had a histologic inflammation after age 60 (vs. 5 in those with histologic inflammation before age 18), 164 cases in those with 0–9 years of education (vs. 47 in those with ≥13 years of education), and 266 cases in those with diabetes before index date (vs. 79 in those without diabetes, Table S10).

### Comparison with earlier literature

Beyond clinical and endoscopic remission [30, 31], histologic remission is increasingly regarded as an aspirational therapeutic target for IBD, although achieving histologic remission remains challenging with current targeted therapies [37] and there is some uncertainty about the definition of histologic remission in CD [31] and UC [30]. Our research has indicated that histologic remission in IBD is associated with a reduced risk of adverse pregnancy outcomes [22], serious infections [20], and infertility [21]. It has been suggested that IBD patients have a higher risk of developing CVD [7, 8, 9, 38] and CVD mortality [12, 39, 40] compared with those without IBD. However, much remains unanswered regarding the link of histologic activity of IBD with CVD. Using Swedish registers, we observed an excess absolute (15/10,000 person‐years) and relative (+16%) risk of MACE after histologic inflammation. Our study reveals for the first time that histologic inflammation increased the risk of myocardial infarction by 29% and heart failure by 19%, even in patients with clinically quiescent IBD (Table S14). However, further research is needed to determine the potential incremental benefits of using histologic remission as a treatment goal over endoscopic remission after considering therapy‐related risks and costs. Moreover, among IBD patients diagnosed more recently (post‐2002 introduction of modern IBD therapy in Sweden), an elevated risk of MACE persisted after histologic inflammation (Table S10), indicating that current IBD care may not sufficiently affect the association between histologic inflammation and MACE.

The observed elevated risk of MACE after clinically active IBD aligns with our expectations and corroborates research suggesting a higher CVD risk during disease active periods. However, those studies applied different surrogates to define disease activity [11, 12, 13, 15, 16]. In studies from Danmark [11, 12, 15], where three markers were used to define disease activity (steroid prescriptions, IBD‐related hospitalization, and initiation of biological treatment), the authors reported an elevated risk of myocardial infarction during flare periods (e.g., rate ratio [RR] = 1.49 [1.16–1.93]) and persistent disease activity (RR = 2.05 [1.58–2.65]) [12]. They also observed a moderately increased risk of stroke during periods of flares (IR ratio [IRR] = 1.57 [1.27–1.93]) and persistent disease activity (IRR = 1.71 [1.32–2.21]), although ischemic stroke was not distinguished from hemorrhagic stroke [11]. Additionally, our finding for heart failure corroborates another Danish study showing heightened heart failure risk during flare (IRR = 2.54 [2.13–3.04]) and persistent activity periods (IRR = 2.73 [2.25–3.33]) [15]. Two UK studies showed an increased risk of myocardial infarction (HR = 1.83 [1.28–2.62], limited to ambulatory patients) [13] during flare periods (defined as within 4 months of receiving a new steroid prescription).

Although the results for clinically active IBD and MACE (e.g., in Table S19) were more pronounced than those for histologic inflammation and MACE (e.g., in Table S10) in our study, direct comparison of risk estimates from the two cohorts should be cautious, given variations in study populations and observation periods.

### Potential mechanisms

Although the underlying mechanisms driving a link between disease activity and MACE remain speculative, potential pathways may involve chronic systemic inflammation [6, 41, 42] and gut dysbiosis [43, 44]. Upregulated pro‐inflammatory cytokines (e.g., tumor necrosis factor‐α and interleukin‐1) induce endothelial dysfunction and plaque formation, activate and aggregate platelets, and lead to atherosclerosis and arterial stiffness [6, 41]. The consequent hemodynamic stress may then promote cardiac remodeling and diastolic dysfunction, potentially leading to heart failure [45]. A shifted gut microbiome promotes pro‐inflammatory cytokine release and reduces short‐chain fatty acids, potentially triggering immune responses and fostering systemic inflammation [44]. The shift also alters bile acid metabolism, affecting lipid absorption and CVD risk [44]. Moreover, patients with IBD are more prone to treatments (i.e., surgery and steroid therapy [17, 38, 46]), which may contribute to the development of MACE. Additionally, the increased risk of venous thromboembolism [38], hypertension [47], and arrhythmias [48, 49] contributes to heart failure in IBD.

### Strengths and limitations

Strengths included the nationwide population‐based study design, large sample size, and virtually complete follow‐up, which provided sufficient power for the prespecified analyses in an unselected population. Moreover, the high PPV for defining IBD and MACE from longitudinally collected registers minimized the risk of misclassification bias. Finally, reporting both absolute and relative risks of MACE facilitates risk communication with patients and health workers.

Several limitations should be acknowledged. First, we did not define histologic activity using a scoring system (e.g., Nancy's Histological index [30]) and lacked data on indications for histologic assessment (e.g., determining severity or estimating the efficacy of treatment), endoscopic quality, macroscopic appearance, and laboratory markers for defining disease activity. Therefore, further studies are needed to validate our definitions of histologic and clinical activity and our findings. Second, our definition of clinical activity, based solely on the health administrative records and largely on steroid use (around 70%), may have predominantly identified those with moderate‐to‐severe disease activity [50]. Consequently, our clinical activity results may be partly driven by the potential raising effect of steroids on CVD risk [38, 51]. Furthermore, we acknowledge that our definition for clinically quiescent IBD may still include patients with clinical or endoscopic activity (e.g., using a 5‐aminosalicylic acid therapy, a drug with a potential cardioprotective effect [38]) or those maintaining the same therapy.

Third, a misclassification of two exposures may arise from several reasons. (a) Histologic assessment may not fully capture disease activity, because the segmental and patchy nature of intestinal inflammation in CD and the diverse microscopic activity within and across colonic segments in UC may be misclassified as histologic remission. (b) Individuals with quiescent IBD may still have ongoing inflammation, given we could not include endoscopic or inflammatory biomarkers when defining clinical activity. (c) Inclusion in the clinical activity study requires residency in Sweden after 2006, as the PDR is available only since July 2005. However, the link between clinically active IBD and MACE in patients diagnosed after 2006 decreases this concern.

Fourth, due to its register‐based nature, residual confounding from unmeasured factors for the exposures (e.g., endoscopic findings) and for MACE (e.g., diet, physical activity, and air pollution) cannot be ruled out, which restricted us from investigating the pure role of histologic/clinical activity and establish causality. Moreover, the impact of smoking cannot be ruled out, despite adjusting for COPD (a proxy for heavy smoking). Fifth, we urge caution when generalizing our findings to other settings because of differences in the incidence of IBD [52, 53] and MACE [54] across countries and regions, as well as Sweden's health care system's provision of nearly free universal access.

### Implications

Our findings of a higher risk of MACE after experiencing histologic inflammation and active IBD support the hypothesis that systemic inflammation increases the risk of atherosclerosis [42, 55], a hypothesis supported by the increased risk of CVDs in other inflammatory diseases (e.g., rheumatoid arthritis) [56]. Meanwhile, our findings have important implications. First, patients and health care providers should be aware of the increased risk of MACE after a record of histologic inflammation and clinically active IBD, especially in patients over 60 years old, those with low educational attainment, and those with CVD‐related comorbidities. For these high‐risk groups, health care professionals should be vigilant for MACE symptoms and proactively assess and manage modifiable MACE risk factors (e.g., hypertension and dyslipidemia) given their high prevalence, following current guideline for CVD risk prevention [57]. Second, refining IBD‐related treatment approaches to lower MACE risk in high‐risk individuals should be considered in clinical practice, particularly in the context that the number of prevalent IBD cases is increasing worldwide [52]. For high‐risk individuals, anti‐inflammatory therapy should be encouraged to decrease the excess risk of MACE, a viewpoint proven by the early success of colchicine in reducing CVD risk in patients with myocardial infarction or coronary artery disease [55]. Besides, prescribing IBD medications with cardiovascular side effects (e.g. steroids and Janus kinase inhibitors [58]) to susceptible individuals warrants careful consideration. Ultimately, our findings, combined with existing evidence on IBD and CVD [38], could be used to develop new guidelines for assessing and managing CVD in IBD patients, especially shortly after IBD diagnosis when the most pronounced IRs for CVD were seen [7, 8, 9].

In conclusion, this nationwide cohort study showed an association between the histologic and clinical activity of IBD and subsequent MACE, including myocardial infarction, ischemic stroke, and heart failure. An increased risk of myocardial infarction and heart failure with histologic inflammation was observed even in patients with clinically quiescent IBD. These findings call for a heightened clinical awareness of MACE in high‐risk populations amidst periods of histologic inflammation and clinically active IBD.

## Author contributions

All authors were involved in the study concept and design. Jiangwei Sun was the guarantor, had access to all the data, did all analyses, and designed figures. Jonas F. Ludvigsson and Ola Olén were involved in the acquisition of data. Jiangwei Sun and Jonas F. Ludvigsson drafted the manuscript and provided the funding. All authors were involved in the interpretation of data and critical revision of the manuscript for important intellectual content and approval of the final version.

## Conflict of interest statement

All authors have completed the ICMJE uniform disclosure form and declare: OO has been PI on projects at Karolinska Institutet financed by grants from Janssen, Pfizer, AbbVie, Takeda, Galapagos/Alfasigma, Ferring, and Bristol Myer Squibb. JH served as speaker or advisory board member for AbbVie, BMS, Celltrion, Eli Lilly, Ferring, Galapagos, Gilead, Index Pharma, Janssen, Medtronic, Merck, MSD, Novartis, Pfizer, Sandoz, Shire, Takeda, Thermo Fisher Scientific, Tillotts Pharma, and Vifor Pharma and received grant support from Takeda, Janssen, and MSD. FE has served as an advisory board member for Boehringer Ingelheim. JFL has coordinated a study for the Swedish IBD Quality Register (SWIBREG), which received funding from Janssen Corporation. JFL has also received financial support from MSD to develop a paper reviewing national health care registers in China and for an unrelated IBD project. JFL also has a research collaboration on celiac disease with Takeda. The other authors report no disclosures relevant to the manuscript.

## Disclosure

The funder of the study had no role in the study design, data collection, data analysis, data interpretation, or writing of the report.

## Ethics statement

This study was approved by the Stockholm Ethics Review Board (2014/1287‐31/4, 2018/972‐32, 2022‐05774‐02, 2021‐06209‐01, 2022‐04384‐02, and 2023‐04868‐02). Individual informed consent was waived as the study was register‐based.

## Supporting information




**Figure S1**: Distribution of histologic inflammation and remission with time since IBD diagnosis. The total number of histologic inflammation periods and remission periods were 99,123 and 46,049, respectively.
**Figure S2**: Distribution of clinically active and quiescent IBD with time since IBD diagnosis. The total number of clinically active IBD periods and quiescent IBD periods were 274,568 and 277,939, respectively.
**Table S1**: ICD codes and SNOMED codes for defining IBD.
**Table S2**: ICD codes for defining phenotypes of IBD.
**Table S3**: SNOMED codes for histologic inflammation and remission.
**Table S4**: IBD‐related surgery.
**Table S5**: ATC codes for IBD medications.
**Table S6**: Definitions of outcomes and comorbidities.
**Table S7**: Baseline characteristics of IBD patients in the cohort of histologic inflammation and histologic remission in 1969–2017.
**Table S8**: Risk of 2‐year MACE for histologic inflammation and histologic remission in patients with IBD.
**Table S9**: Risk of 2‐year MACE for histologic inflammation and histologic remission in patients with CD, UC, and IBD‐U.
**Table S10**: Subgroup analyses for risk of 2‐year MACE for histologic inflammation and histologic remission in patients with IBD.
**Table S11**: Risk of 2‐year MACE for histologic inflammation and histologic remission in patients with IBD, stratified by the phenotypes of the Montreal Classification.
**Table S12**: Risk of 5‐year MACE for histologic inflammation and histologic remission in patients with IBD.
**Table S13**: Risk of 2‐year MACE for histologic inflammation and histologic remission in patients with IBD.
**Table S14**: Risk of 2‐year MACE for histologic inflammation and histologic remission during periods of clinically quiescent IBD.
**Table S15**: Sensitivity analyses of the 2‐year MACE risk for histologic inflammation and histologic remission in patients with IBD.
**Table S16**: Baseline characteristics of IBD patients in the cohort of clinically active and quiescent IBD in 2006–2020.
**Table S17**: Risk of 2‐year MACE for clinically active and quiescent IBD in patients with IBD.
**Table S18**: Risk of 2‐year MACE for clinically active and quiescent IBD in patients with CD, UC, and IBD‐U.
**Table S19**: Subgroup analyses for risk of 2‐year MACE for clinically active and quiescent IBD in patients with IBD.
**Table S20**: Risk of 2‐year MACE for clinically active and quiescent IBD in patients with IBD, stratified by the phenotypes of the Montreal Classification.
**Table S21**: Risk of 5‐year MACE for clinically active and quiescent IBD in patients with IBD.
**Table S22**: Risk of 2‐year MACE for clinically active and quiescent IBD in patients with IBD.

## Data Availability

Under current data protection legislation, the data cannot be shared directly and must be requested from the respective registry holders, Statistics Sweden (information@scb.se) and the Swedish National Board of Health and Welfare (registerservice@socialstyrelsen.se), after approval by the Swedish Ethical Review Authority.
