# Supplementary material for: Association of histologic and clinical activity with major adverse cardiovascular events in patients with inflammatory bowel disease: A cohort study
Source: J Intern Med. 2025 Oct 23;298(6):697–710. doi: 10.1111/joim.70035 (PMC12617494; doi:10.1111/joim.70035)
Supplement: Supplementary file 1 — Figure S1: Distribution of histologic inflammation and remission with time since IBD diagnosis. The total number of histologic inflammation periods and remission periods were 99,123 and 46,049, respectively. Figure S2: Distribution of clinically active and quiescent IBD with time since IBD diagnosis. The total number of clinically active IBD periods and quiescent IBD periods were 274,568 and 277,939, respectively. Table S1: ICD codes and SNOMED codes for defining IBD. Table S2: ICD codes for defining phenotypes of IBD. Table S3: SNOMED codes for histologic inflammation and remission. Table S4: IBD‐related surgery. Table S5: ATC codes for IBD medications. Table S6: Definitions of outcomes and comorbidities. Table S7: Baseline characteristics of IBD patients in the cohort of histologic inflammation and histologic remission in 1969–2017. Table S8: Risk of 2‐year MACE for histologic inflammation and histologic remission in patients with IBD. Table S9: Risk of 2‐year MACE for histologic inflammation and histologic remission in patients with CD, UC, and IBD‐U. Table S10: Subgroup analyses for risk of 2‐year MACE for histologic inflammation and histologic remission in patients with IBD. Table S11: Risk of 2‐year MACE for histologic inflammation and histologic remission in patients with IBD, stratified by the phenotypes of the Montreal Classification. Table S12: Risk of 5‐year MACE for histologic inflammation and histologic remission in patients with IBD. Table S13: Risk of 2‐year MACE for histologic inflammation and histologic remission in patients with IBD. Table S14: Risk of 2‐year MACE for histologic inflammation and histologic remission during periods of clinically quiescent IBD. Table S15: Sensitivity analyses of the 2‐year MACE risk for histologic inflammation and histologic remission in patients with IBD. Table S16: Baseline characteristics of IBD patients in the cohort of clinically active and quiescent IBD in 2006–2020. Table S17: Risk of 2‐year MACE for clinicall [file JOIM-298-697-s001.docx]

**Association of histologic and clinical activity with major adverse cardiovascular events in patients with inflammatory bowel disease: A cohort study**

Jiangwei Sun, PhD ^1^, Karl Mårild, MD, PhD ^2,3^, Johan Sundström MD, PhD ^4, 5^, David Bergman, MD, PhD ^1^, SWIBREG Study Group**,* Fahim Ebrahimi, MD, MSc ^1,6^, Jonas Halfvarson, MD, PhD ^7^, Ola Olén, MD, PhD ^8,9^, Jonas F. Ludvigsson, MD, PhD ^1,10,11^

**Collaborators: Hans Strid, Henrik Hjortswang, Malin Olsson, Johann Hreinsson, Charlotte Hedin, Jonas L Bengtsson, Marie A Andersson, Pontus Karling, Martin Rejler, Susanna Jäghult, Ulrika Fagerberg, Pär Myrelid, Caroline Nordenvall*

^1^ Department of Medical Epidemiology and Biostatistics, Karolinska Institutet, Stockholm, Sweden

^2^ Department of Pediatrics, Institute of Clinical Sciences, Sahlgrenska Academy, Gothenburg, Sweden

^3^ Department of Pediatrics, Queen Silvia Children’s Hospital, Gothenburg, Sweden

^4^ Department of Medical Sciences, Uppsala University, Sweden

^5^ The George Institute for Global Health, University of New South Wales, Australia

^6^ Department of Gastroenterology and Hepatology, University Digestive Health Care Center Basel – Clarunis, Basel, Switzerland

^7^ Department of Gastroenterology, Faculty of Medicine and Health, Örebro University, Örebro, Sweden

^8^ Division of Clinical Epidemiology, Department of Medicine Solna, Karolinska Institutet, Stockholm, Sweden

^9^ Sachs’ Children and Youth Hospital, Stockholm South General Hospital, Stockholm, Sweden

^10^ Department of Pediatrics, Örebro University Hospital, Örebro, Sweden

^11^ Division of Digestive and Liver Disease, Department of Medicine, Columbia University

Medical Center, New York, USA

Contents

[eMethods 1](#_Toc195622162)

[Figure S1: Distribution of histologic inflammation and remission with time since IBD diagnosis. The total number of histologic inflammation periods and remission periods were 99,123 and 46,049, respectively. 2](#_Toc195622163)

[Figure S2: Distribution of clinically active and quiescent IBD with time since IBD diagnosis. The total number of clinically active IBD periods and quiescent IBD periods were 274,568 and 277,939, respectively. 3](#_Toc195622164)

[Table S1. ICD codes and SNOMED codes for defining IBD 4](#_Toc195622165)

[Table S2. ICD codes for defining phenotypes of IBD 5](#_Toc195622166)

[Table S3. SNOMED codes for histologic inflammation and remission 6](#_Toc195622167)

[Table S4. IBD-related surgery 7](#_Toc195622168)

[Table S5. ATC codes for IBD medications 8](#_Toc195622169)

[Table S6. Definitions of outcomes and comorbidities 9](#_Toc195622170)

[Table S7. Baseline characteristics of IBD patients in the cohort of histologic inflammation and histologic remission in 1969– 2017 10](#_Toc195622171)

[Table S8. Risk of 2-year MACE for histologic inflammation and histologic remission in patients with IBD 12](#_Toc195622172)

[Table S9. Risk of 2-year MACE for histologic inflammation and histologic remission in patients with CD, UC, and IBD-U 13](#_Toc195622173)

[Table S10. Subgroup analyses for risk of 2-year MACE for histologic inflammation and histologic remission in patients with IBD 14](#_Toc195622174)

[Table S11. Risk of 2-year MACE for histologic inflammation and histologic remission in patients with IBD, stratified by the phenotypes of the Montreal Classification 17](#_Toc195622175)

[Table S12. Risk of 5-year MACE for histologic inflammation and histologic remission in patients with IBD 18](#_Toc195622176)

[Table S13. Risk of 2-year MACE for histologic inflammation and histologic remission in patients with IBD ^a^ 19](#_Toc195622177)

[Table S14. Risk of 2-year MACE for histologic inflammation and histologic remission during periods of clinically quiescent IBD 20](#_Toc195622178)

[Table S15. Sensitivity analyses of the 2-year MACE risk for histologic inflammation and histologic remission in patients with IBD 21](#_Toc195622179)

[Table S16. Baseline characteristics of IBD patients in the cohort of clinically active and quiescent IBD in 2006–2020 22](#_Toc195622180)

[Table S17. Risk of 2-year MACE for clinically active and quiescent IBD in patients with IBD 24](#_Toc195622181)

[Table S18. Risk of 2-year MACE for clinically active and quiescent IBD in patients with CD, UC, and IBD-U 25](#_Toc195622182)

[Table S19. Subgroup analyses for risk of 2-year MACE for clinically active and quiescent IBD in patients with IBD 26](#_Toc195622183)

[Table S20. Risk of 2-year MACE for clinically active and quiescent IBD in patients with IBD, stratified by the phenotypes of the Montreal Classification 28](#_Toc195622184)

[Table S21. Risk of 5-year MACE for clinically active and quiescent IBD in patients with IBD 29](#_Toc195622185)

[Table S22. Risk of 2-year MACE for clinically active and quiescent IBD in patients with IBD ^a^ 30](#_Toc195622186)

[Reference 31](#_Toc195622187)

eMethods

Data source

This study was based on nationwide Swedish healthcare registers (e.g. National Patient Register [NPR]^1^, Prescribed Drug Register [PDR]^2^, Total Population Register^3^, and Cause of Death Register^4^), the nationwide histopathology cohort ESPRESSO (Epidemiology Strengthened by histoPathology Reports in Sweden)^5^, and the Swedish Inflammatory Bowel Disease Register (SWIBREG)^6^.

The NPR was founded in 1964 and covers nationwide inpatient care since 1987 and outpatient care since 2001. The PDR covers information on all prescribed drugs dispensed in Sweden since July 2005. Death date was retrieved from the Total Population Register (since 1968) and death cause was identified from the Cause of Death Register (since 1952). The ESPRESSO cohort includes individuals with gastrointestinal biopsies from all 28 pathology departments in Sweden from 1965 to 2017. The SWIBREG (since 2005) is a quality register for IBD in Sweden.

Covariates

In addition to age at IBD diagnosis, sex, calendar year at index date, and county of residence, we also considered the following covariates. Educational attainment was identified from the Swedish Longitudinal Integrated Database for Health Insurance and Labor Market Studies (available since 1990)^7^ with four categories: 0-9 years, 10-12 years, ≥13 years, and “missing”. Country of birth (Nordic [including Sweden, Denmark, Finland, Norway, and Iceland] or others) was identified from the TPR^3^. Number of health care visits between 2 years and 6 months before the index date (0, 1, 2-3, and ≥4), a proxy for regular healthcare seeking behavior was retrieved from the NPR. Finally, from the NPR, we also considered comorbidities before the index date, including hypertension, diabetes, obesity, dyslipidemia, chronic kidney disease, and chronic obstructive pulmonary disease (COPD, a proxy for heavy smoking, only if the patient was diagnosed ≥40 years of age).

Except for age at IBD diagnosis, sex, and country of birth, the other covariates were updated at the index date (i.e., time-varying covariates).


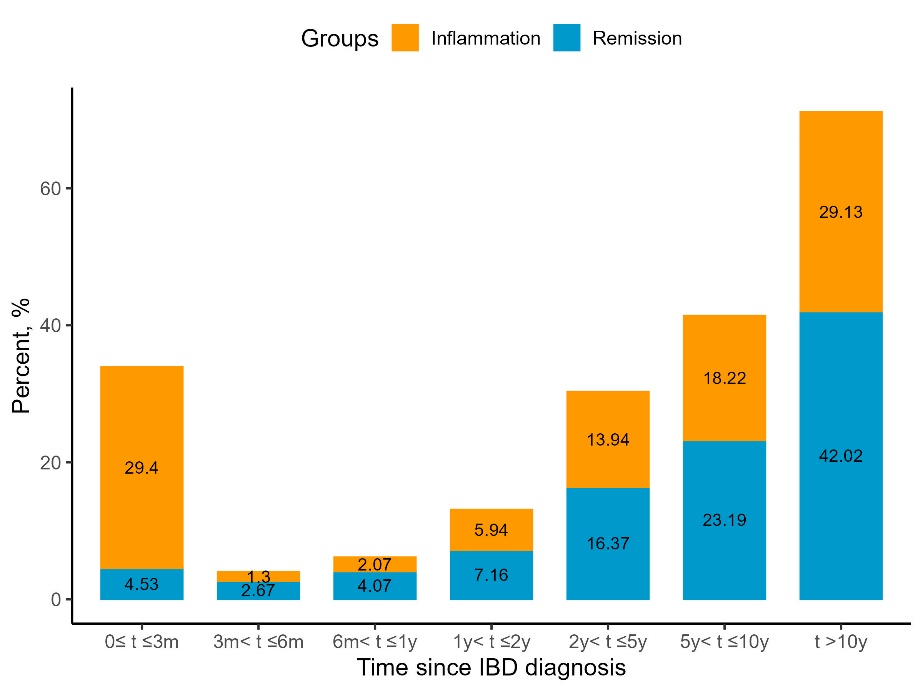


Figure S1: Distribution of histologic inflammation and remission with time since IBD diagnosis. The total number of histologic inflammation periods and remission periods were 99,123 and 46,049, respectively.


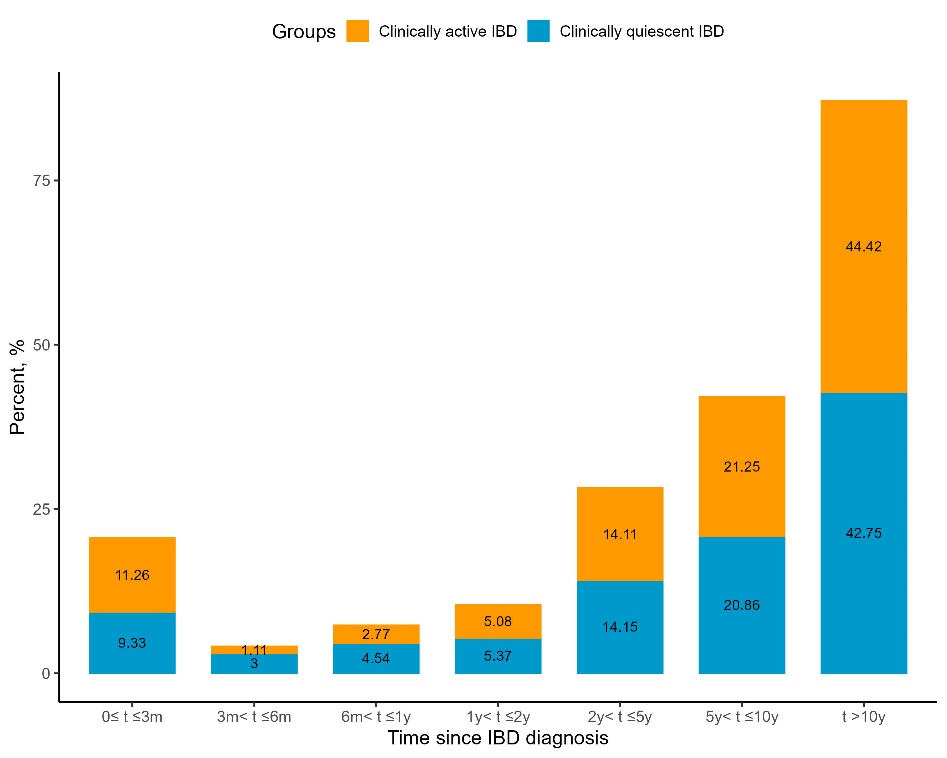


Figure S2: Distribution of clinically active and quiescent IBD with time since IBD diagnosis. The total number of clinically active IBD periods and quiescent IBD periods were 274,568 and 277,939, respectively.

Table S1. ICD codes and SNOMED codes for defining IBD

| IBD subtypes ^a^ | ICD-7  (1964-1968) | ICD-8  (1969-1986) | ICD-9  (1987-1996) | ICD-10  (1997-) | SNOMED codes ^b^ |
| --- | --- | --- | --- | --- | --- |
| UC | 572,20; 572,21; 578,03 | 563,1; 563,10; 569,02; 569,04 | 556 | K51 | D6255 or M41; M42; M43; M44; M463; or M47 |
| CD | 572,00; 572,09 | 563,00 | 555 | K50 | D6216 or M41; M42; M43; M44; M463; or M47 |
| IBD-U | UC + CD | UC + CD or 563; 563,0; 563,9; 563,98; 563,99 | UC + CD | UC + CD or K523 | D6214 or M41; M42; M43; M44; M463; or M47 |
| CD: Crohn’s disease; IBD(-U): inflammatory bowel disease (unclassified); ICD: International Classification of Disease; SNOMED: Systematized Nomenclature of Medicine; UC: ulcerative colitis.  ^a^ Diagnosis criteria: having either ≥2 ICD codes in the National Patient Register or ≥1 ICD code for IBD **AND** ≥1 SNOMED code for IBD.  ^b^ In SNOMED codes, D codes are the diagnostic codes, D6255 for example was the diagnostic code for UC. Meanwhile, codes starting with “M” (one unspecific SNOMED code) refer to all codes starting with the respective code.  Subtypes of IBD were defined according to the first ICD and SNOMED codes only (i.e., no information after start of follow-up contributed to the subtype definition); in case one individual had one ICD code for IBD and one unspecific SNOMED code (i.e., the "M" code), the IBD subtype was solely determined by the ICD code. | | | | | |

Table S2. ICD codes for defining phenotypes of IBD

| Montreal classification | Diagnostic codes |
| --- | --- |
| CD location ^a^ |  |
| Ileal (L1) | K500 |
| Colonic (L2) | K501 |
| Ileocolonic or location not defined (L3/LX) | K508; K509 |
| Perianal disease modifier | Any of the diagnostic codes: K603; K604; K605; K610; K611; K612; K613; K614; K624 OR any of the surgical procedure codes: JHD20; JHD30; JHD33; JHD50; JHD60; JHD63; JHA00; JHA20; JHW96 |
| UC extent ^a^ |  |
| Proctitis (E1) | K512 |
| Left-sided colitis (E2) | K513; K515 |
| Extensive colitis (E3) | K510 |
| Extent not defined (EX) | K514; K518; K519 |
| Primary sclerosing cholangitis ^b^ | ICD-9 (1987-1996): 576B |
|  | ICD-10 (1997-): K830 |
| Other extraintestinal manifestations ^b^ | ICD-9: 695C; 364; 713B; 720A; 720C; 720W; 720X |
|  | ICD-10: L52; L88; L982; H20; M074; M075; M076; |
|  | M091; M092; M45; M460; M461; M468; M469; M139; M255 |

CD: Crohn’s disease; E: extent; IBD(-U): inflammatory bowel disease (unclassified); ICD: International Classification of Disease; L: location; UC: ulcerative colitis.

^a^ The Montreal classification for defining disease phenotypes was available since the use of ICD-10 in Sweden (1997-). All codes are captured in the Swedish National Patient Register (prospectively recorded in routine clinical practice).

In this study, we categorized Crohn's disease location into two groups: ileal (L1)/ileocolonic (L3)/unknown (LX) or colonic (L2), and categorized ulcerative colitis extent into three groups: proctitis (E1)/left-sided colitis (E2), extensive colitis (E3) or extent not defined (EX).

^b^ We restricted our use to ICD-9 and ICD-10 codes since the earlier ICD codes for extraintestinal inflammation are less reliable, particularly for primary sclerosing cholangitis. The validity of the PSC codes has not formally been tested in Sweden^8^.

| Table S3. SNOMED codes for histologic inflammation and remission | |
| --- | --- |
| SNOMED code | Morphology |
| M00100 | Normal mucosa |
| M00110 | Normal mucosa |
| M40000 | Unspecified inflammation |
| M40400 | Pustulous inflammation, unspecified |
| M41000 | Acute inflammation |
| M42100 | Acute and chronic inflammation |
| M43000 | Chronic inflammation |
| M43030 | Ulcer, chronic inflammation |
| M42000 | Subacute inflammation |
| M40460 | Inflammation with pus |
| M40700 | Inflammation with necrosis |
| M41700 | Inflammation with necrosis, abscess |
| M41740 | Abscess |
| M44000 | Granulomatous inflammation |
| M44700 | Necrotizing granulomatous inflammation |
| M44900 | Necrotizing fibrinous inflammation |
| M45020 | Inflammation with granulation |
| SNOMED: the Systematized Nomenclature of Medicine code | |

| Table S4. IBD-related surgery | | | | |
| --- | --- | --- | --- | --- |
| Surgery procedure | Surgery code (1963 -1996) | Surgery code (1997-) | Apply to subtype of IBD | Exclude if colorectal cancer +/- 1 month |
| Colectomy with ileostomy | 4651 | JFH10 | UC + CD | Colectomy |
| Laparoscopic colectomy with ileostomy | - | JFH11 | UC + CD | Colectomy |
| Other colectomy | - | JFH96 | UC + CD, if with stoma formation code | Colectomy |
| Formation of a loopileostomy | 0054 | JFF10 | CD | Stoma |
| Laparoscopic formation of a loopileostomy | - | JFF11 | CD | Stoma |
| Formation of enterostoma | - | JFF13 | CD | Stoma |
| Other formation of stoma on small bowel or colon | 0054 | JFF96 | CD | Stoma |
| Other laparoscopic formation of stoma on small bowel or colon | - | JFF97 | CD | Stoma |
| Ileocecal resection | 4642 | JFB20 | CD, if with stoma formation code | Ileocecal resection |
| Laparoscopic ileocecal resection | - | JFB21 | CD, if with stoma formation code | Ileocecal resection |
| Right-sided colectomy | 4641 | JFB30 | CD, if with stoma formation code | Hemi-colectomy |
| Laparoscopic right-sided colectomy | - | JFB31 | CD, if with stoma formation code | Hemi-colectomy |
| CD: Crohn’s disease; IBD: inflammatory bowel disease; UC: ulcerative colitis. | | | | |

| Table S5. ATC codes for IBD medications | |
| --- | --- |
| Medications | ATC code ^a^ |
| Immune modulators |  |
| Azathioprine | L04AX01 |
| Mercaptopurine | L01BB02 |
| Methotrexate | L04AX03 |
| Anti–tumor necrosis factor alpha |  |
| Infliximab | L04AB02 (L04AA12 before 2008) |
| Adalimumab | L04AB04 (L04AA17 before 2008) |
| Golimumab | L04AB06 |
| Other biologics/small molecules |  |
| Vedolizumab | L04AA33/L04AG05 |
| Ustekinumab | L04AC05 |
| Risankizumab | L04AC18 |
| Mirikizumab | L04AC24 |
| Tofacitinib | L04AA29/L04AF01 |
| Ozanimod | L04AA38/L04AE02 |
| Etrazimod | L04AE05 |
| Filgotinib | L04AA45/L04AF04 |
| Upadacitinib | L04AA44/L04AF03 |
| Systemic corticosteroids |  |
| Betamethasone | H02AB01 |
| Methylprednisolone | H02AB04 |
| Prednisolone | H02AB06 |
| Prednisone | H02AB07 |
| Cortisone | H02AB10 |
| Oral corticosteroids acting locally |  |
| Budesonide | A07EA06 |

ATC: Anatomical Therapeutic Chemical; IBD: inflammatory bowel disease

^a^ Commercial names were also used to identify biologics from SWIBREG: infliximab (Remicade, Remsima, Inflectra, Flixabi, Zessly); adalimumab (Humira, Amgevita, Imraldi, Cyltezo, Hulio, Hyrimoz, Idacio); golimumab (Simponi); vedolizumab (Entyvio); ustekinumab (Stelara); tofacitinib (Xeljanz); ozanimod (Zeposia); filgotinib (Jyseleca); risankizumab (Skyrizi), mirikizumab (Omvoh), etrazimod (Velsipity).

| Table S6. Definitions of outcomes and comorbidities | | | |
| --- | --- | --- | --- |
|  | ICD-8 code | ICD-9 code | ICD-10 code |
| **Outcomes** |  |  |  |
| Major adverse cardiovascular event | A composite outcome including ischemic heart disease, stroke, and heart failure | | |
| Ischemic heart disease | 410-411; 412-414 | 410; 411-414 | I20; I21-I22; I23-I25 |
| Myocardial infarction | 410-411; 412,01; 412,91 | 410 | I21-I22 |
| Stroke |  |  |  |
| Hemorrhagic stroke | 430; 431 | 430; 431 | I60; I61 |
| Ischemic stroke | 432; 433; 434 | 433; 434 | I63 |
| Heart failure | 427,00; 427,10; 428,99 | 428 | I50; I110 |
| **CVD-related comorbidities** |  |  |  |
| Hypertension | 400-404 | 401-405 | I10; I119; I12-I15 |
| Obesity | 277 | 278A; 278B | E65-E66 |
| Diabetes | 250 | 250 | E10-E14; O24 |
| Dyslipidemia | 279 | 272 | E78 |
| Chronic kidney disease | 585; 586; Y29,01 | 585; 586; 753B; V42A; V45B; V56 | N18; N19; N26; T824; Y841; Q61; Z49; Z992; Z940 |
| Chronic obstructive pulmonary disease (COPD, only if patient diagnosed ≥40 year) | COPD: 491, 492 | COPD: 491, 492, 496 | COPD: J41-J44 |
| ICD: International Classification of Diseases. | | | |
| The ICD codes of stroke in the above table were used to exclude those with prevalence stroke before index date, but 433 in ICD9 and 432 in ICD8 were not considered to identify the newly diagnosed stroke. | | | |

| Table S7. Baseline characteristics of IBD patients in the cohort of histologic inflammation and histologic remission in 1969– 2017 | | | | |
| --- | --- | --- | --- | --- |
|  | Patients with IBD | Subtypes of IBD | | |
|  |  | CD | UC | IBD-U |
| No. of patients | 59168 | 17093 | 38877 | 3198 |
| Age at IBD diagnosis, years |  |  |  |  |
| Mean ± SD | 38.8 ± 17.9 | 35.7 ± 17.5 | 40.1 ± 17.8 | 39.3 ± 19.6 |
| Median (IQR) | 36.2 (24.4-51.7) | 32.1 (21.6-48.1) | 37.9 (26.0-52.9) | 36.0 (22.7-53.9) |
| <18 | 6754 (11.4) | 2694 (15.8) | 3594 (9.2) | 466 (14.6) |
| 18-<40 | 26854 (45.4) | 8149 (47.7) | 17371 (44.7) | 1334 (41.7) |
| 40-<60 | 16914 (28.6) | 4331 (25.3) | 11765 (30.3) | 818 (25.6) |
| ≥60 | 8646 (14.6) | 1919 (11.2) | 6147 (15.8) | 580 (18.1) |
| Female | 28455 (48.1) | 8799 (51.5) | 18077 (46.5) | 1579 (49.4) |
| Born in Nordic country | 54920 (92.8) | 15719 (92.0) | 36209 (93.1) | 2992 (93.6) |
| Calendar period at IBD diagnosis |  |  |  |  |
| 1969-1989 | 8563 (14.5) | 2968 (17.4) | 4845 (12.5) | 750 (23.5) |
| 1990-2001 | 18659 (31.5) | 5345 (31.3) | 12831 (33.0) | 483 (15.1) |
| 2002-2009 | 18628 (31.5) | 5212 (30.5) | 12775 (32.9) | 641 (20.0) |
| 2010-2017 | 13318 (22.5) | 3568 (20.9) | 8426 (21.7) | 1324 (41.4) |
| Educational attainment |  |  |  |  |
| 0-9 y | 12420 (21.0) | 3725 (21.8) | 7985 (20.5) | 710 (22.2) |
| 10-12 y | 23801 (40.2) | 6735 (39.4) | 15897 (40.9) | 1169 (36.6) |
| ≥13 y | 14344 (24.2) | 3694 (21.6) | 9998 (25.7) | 652 (20.4) |
| Missing | 8603 (14.5) | 2939 (17.2) | 4997 (12.9) | 667 (20.9) |
| Montreal Classification CD at IBD diagnosis |  |  |  |  |
| No location information | - | 5555 (32.5) | - | - |
| L1, L3/LX (Ileal, ileocolonic or location not defined) | - | 8733 (51.1) | - | - |
| L2 (Colonic) | - | 2805 (16.4) | - | - |
| Perianal | - | 816 (4.8) | - | - |
| Montreal Classification UC at IBD diagnosis |  |  |  |  |
| No extent information ^a^ | - | - | 10378 (26.7) | - |
| E1/E2 (Proctitis, left-sided colitis) | - | - | 10456 (26.9) | - |
| E3 (Extensive colitis) | - | - | 5711 (14.7) | - |
| EX (Extent not defined) | - | - | 12332 (31.7) | - |
| Extraintestinal manifestations at IBD diagnosis |  |  |  |  |
| Primary sclerosing cholangitis | 479 (0.8) | 70 (0.4) | 395 (1.0) | 14 (0.4) |
| Other extraintestinal manifestations | 1803 (3.1) | 662 (3.9) | 981 (2.5) | 160 (5.0) |
| CD: Crohn’s disease; IBD(-U): inflammatory bowel disease (unclassified); IQR: interquartile range; SD: standard deviation; UC: ulcerative colitis. | | | | |
| ^a^ No extent information refers to patients who were diagnosed with UC before the introduction of ICD-10 in 1997. | | | | |

| Table S8. Risk of 2-year MACE for histologic inflammation and histologic remission in patients with IBD | | | | | |
| --- | --- | --- | --- | --- | --- |
| Outcomes | No. of events (IR, per 10,000 person-years) | | IR difference (95%CIs), per 10,000 person-years | HR (95%CIs) | |
|  | Histologic inflammation | Histologic remission |  | Model 1 ^a^ | Model 2 ^b^ |
| MACE | 868 (86.3) | 558 (71.3) | 15.1 (6.8 to 23.3) | 1.17 (1.05 to 1.31) | 1.16 (1.04 to 1.30) |
| Ischemic heart disease | 528 (51.4) | 331 (41.4) | 10.0 (3.7 to 16.2) | 1.17 (1.01 to 1.35) | 1.16 (1.00 to 1.34) |
| Myocardial infarction | 334 (31.8) | 182 (22.2) | 9.6 (4.9 to 14.3) | 1.35 (1.12 to 1.63) | 1.36 (1.12 to 1.64) |
| Stroke | 319 (30.2) | 193 (23.5) | 6.7 (2.0 to 11.4) | 1.24 (1.03 to 1.49) | 1.21 (1.00 to 1.46) |
| Hemorrhagic stroke | 63 (5.9) | 39 (4.7) | 1.2 (-0.9 to 3.3) | 1.29 (0.85 to 1.95) | 1.22 (0.80 to 1.85) |
| Ischemic stroke | 250 (23.5) | 146 (17.7) | 5.9 (1.8 to 10.0) | 1.32 (1.07 to 1.63) | 1.31 (1.06 to 1.62) |
| Heart failure | 466 (44.0) | 238 (28.9) | 15.1 (9.7 to 20.6) | 1.36 (1.16 to 1.60) | 1.35 (1.15 to 1.60) |
| CI: confidence interval; HR: hazard ratio; IR: incidence rate; MACE: major adverse cardiovascular events. | | | | | |
| ^a^ Adjusted for age at IBD diagnosis, sex, and calendar year at index date. | | | | | |
| ^b^ Further adjusted for county of residence, educational attainment, country of birth, number of health care visits, hypertension, diabetes, obesity, dyslipidemia, chronic kidney disease, and COPD. | | | | | |

| Table S9. Risk of 2-year MACE for histologic inflammation and histologic remission in patients with CD, UC, and IBD-U | | | | | |
| --- | --- | --- | --- | --- | --- |
| Outcomes | No. of events (IR, per 10,000 person-years) | | IR difference (95%CIs), per 10,000 person-years | HR (95%CIs) | |
|  | Histologic inflammation | Histologic remission |  | Model 1 ^a^ | Model 2 ^b^ |
|  | Patients with CD | | | | |
| MACE | 197 (77.4) | 129 (58.8) | 18.6 (3.8 to 33.4) | 1.33 (1.05 to 1.67) | 1.30 (1.03 to 1.64) |
| Ischemic heart disease | 105 (40.4) | 67 (29.9) | 10.6 (0.0 to 21.1) | 1.31 (0.95 to 1.80) | 1.33 (0.97 to 1.84) |
| Myocardial infarction | 63 (23.8) | 38 (16.5) | 7.3 (-0.6 to 15.2) | 1.41 (0.93 to 2.15) | 1.37 (0.90 to 2.09) |
| Stroke | 67 (25.3) | 50 (21.8) | 3.5 (-5.1 to 12.1) | 1.19 (0.81 to 1.73) | 1.15 (0.79 to 1.69) |
| Hemorrhagic stroke | 14 (5.2) | 17 (7.3) | -2.1 (-6.5 to 2.3) | 0.73 (0.35 to 1.54) | 0.64 (0.30 to 1.39) |
| Ischemic stroke | 54 (20.3) | 31 (13.5) | 6.9 (-0.4 to 14.1) | 1.53 (0.97 to 2.41) | 1.51 (0.96 to 2.39) |
| Heart failure | 108 (40.9) | 63 (27.4) | 13.4 (3.2 to 23.7) | 1.39 (1.00 to 1.92) | 1.39 (1.00 to 1.94) |
|  | Patients with UC | | | | |
| MACE | 611 (87.9) | 399 (76.1) | 11.7 (1.5 to 21.9) | 1.17 (1.05 to 1.31) | 1.13 (1.01 to 1.27) |
| Ischemic heart disease | 388 (54.6) | 247 (46.2) | 8.4 (0.5 to 16.3) | 1.11 (0.94 to 1.31) | 1.09 (0.92 to 1.29) |
| Myocardial infarction | 246 (33.8) | 133 (24.2) | 9.6 (3.7 to 15.5) | 1.30 (1.04 to 1.62) | 1.31 (1.05 to 1.64) |
| Stroke | 226 (30.8) | 136 (24.6) | 6.2 (0.4 to 11.9) | 1.18 (0.94 to 1.47) | 1.16 (0.93 to 1.46) |
| Hemorrhagic stroke | 44 (5.9) | 22 (3.9) | 2.0 (-0.4 to 4.4) | 1.55 (0.91 to 2.63) | 1.51 (0.89 to 2.58) |
| Ischemic stroke | 172 (23.3) | 109 (19.7) | 3.7 (-1.4 to 8.7) | 1.17 (0.91 to 1.51) | 1.18 (0.91 to 1.51) |
| Heart failure | 319 (43.4) | 155 (28.0) | 15.4 (8.9 to 21.8) | 1.39 (1.13 to 1.69) | 1.37 (1.12 to 1.68) |
|  | Patients with IBD-U | | | | |
| MACE | 60 (108.0) | 30 (75.9) | 32.1 (-6.5 to 70.6) | 1.42 (0.89 to 2.26) | 1.37 (0.85 to 2.21) |
| Ischemic heart disease | 35 (61.2) | 17 (42.2) | 19.0 (-9.6 to 47.5) | 1.40 (0.76 to 2.55) | 1.27 (0.68 to 2.35) |
| Myocardial infarction | 25 (42.6) | 11 (26.5) | 16.1 (-6.8 to 39.0) | 1.66 (0.78 to 3.50) | 1.48 (0.69 to 3.18) |
| Stroke | 26 (44.3) | 7 (16.8) | 27.5 (6.4 to 48.6) | 2.64 (1.12 to 6.24) | 3.26 (1.31 to 8.10) |
| Hemorrhagic stroke | 5 (8.3) | 0 (0.0) | 8.3 (1.0 to 15.6) | NA | NA |
| Ischemic stroke | 24 (40.7) | 6 (14.4) | 26.3 (6.4 to 46.2) | 2.75 (1.09 to 6.93) | 2.97 (1.14 to 7.73) |
| Heart failure | 39 (66.0) | 20 (47.9) | 18.1 (-11.4 to 47.6) | 1.26 (0.71 to 2.24) | 1.34 (0.74 to 2.45) |
| CI: confidence interval; HR: hazard ratio; IR: incidence rate; MACE: major adverse cardiovascular events; NA: not available. | | | | | |
| ^a^ Adjusted for age at IBD diagnosis, sex, and calendar year at index date. | | | | | |
| ^b^ Further adjusted for county of residence, educational attainment, country of birth, number of health care visits, hypertension, diabetes, obesity, dyslipidemia, chronic kidney disease, and COPD. | | | | | |

| Table S10. Subgroup analyses for risk of 2-year MACE for histologic inflammation and histologic remission in patients with IBD | | | | | |
| --- | --- | --- | --- | --- | --- |
| Outcomes | No. of events (IR, per 10,000 person-years) | | IR difference (95%CIs), per 10,000 person-years | HR (95%CIs) | |
|  | Histologic inflammation | Histologic remission |  | Model 1 ^a^ | Model 2 ^b^ |
| Sex |  | | | | |
| Male | 519 (96.7) | 340 (84.0) | 12.7 (0.5 to 24.9) | 1.14 (0.99 to 1.32) | 1.12 (0.97 to 1.29) |
| Female | 349 (74.5) | 218 (57.6) | 16.8 (5.9 to 27.8) | 1.22 (1.02 to 1.45) | 1.21 (1.02 to 1.45) |
| Age at IBD diagnosis, years |  |  |  |  |  |
| <18 | 13 (10.0) | 19 (18.6) | -8.6 (-18.5 to 1.4) | 0.71 (0.35 to 1.47) | 0.70 (0.34 to 1.47) |
| 18-<40 | 98 (19.9) | 109 (28.7) | -8.8 (-15.4 to -2.1) | 0.96 (0.73 to 1.28) | 0.93 (0.70 to 1.23) |
| 40-<60 | 268 (96.2) | 247 (101.7) | -5.5 (-22.7 to 11.6) | 1.09 (0.91 to 1.30) | 1.05 (0.88 to 1.26) |
| ≥60 | 489 (463.4) | 183 (315.6) | 147.8 (86.3 to 209.3) | 1.38 (1.16 to 1.63) | 1.37 (1.15 to 1.63) |
| Age at index date, years |  |  |  |  |  |
| <18 | 3 (4.9) | 1 (2.7) | 2.2 (-5.6 to 9.9) | 1.67 (0.17 to 16.17) | 1.69 (0.17 to 16.33) |
| 18-<40 | 30 (7.7) | 24 (9.2) | -1.5 (-6.1 to 3.1) | 0.89 (0.51 to 1.55) | 0.95 (0.55 to 1.66) |
| 40-<60 | 204 (55.2) | 167 (51.5) | 3.7 (-7.2 to 14.6) | 1.06 (0.86 to 1.31) | 1.00 (0.81 to 1.24) |
| ≥60 | 631 (337.2) | 366 (226.0) | 111.2 (76.1 to 146.2) | 1.23 (1.07 to 1.40) | 1.22 (1.07 to 1.40) |
| Calendar period at IBD diagnosis | |  |  |  |  |
| 1969-1989 | 192 (76.5) | 153 (97.3) | -20.8 (-39.6 to -2.0) | 0.92 (0.73 to 1.15) | 0.92 (0.73 to 1.15) |
| 1990-2001 | 312 (83.2) | 216 (71.7) | 11.5 (-1.8 to 24.8) | 1.06 (0.88 to 1.27) | 1.08 (0.89 to 1.30) |
| 2002-2009 | 234 (92.7) | 148 (62.2) | 30.6 (15.0 to 46.1) | 1.32 (1.06 to 1.63) | 1.24 (1.00 to 1.54) |
| 2010-2017 | 130 (102.3) | 41 (47.5) | 54.9 (32.1 to 77.7) | 1.73 (1.21 to 2.46) | 1.83 (1.28 to 2.61) |
| Calendar period at index date |  |  |  |  |  |
| 1969-1989 | 41 (62.9) | 9 (50.1) | 12.8 (-25.1 to 50.8) | 0.87 (0.42 to 1.82) | 0.99 (0.46 to 2.13) |
| 1990-2001 | 273 (92.5) | 82 (66.7) | 25.8 (7.7 to 44.0) | 1.22 (0.95 to 1.57) | 1.29 (1.00 to 1.66) |
| 2002-2009 | 266 (84.7) | 195 (76.2) | 8.5 (-6.3 to 23.2) | 1.06 (0.88 to 1.28) | 1.00 (0.82 to 1.21) |
| 2010-2021 | 288 (87.0) | 272 (70.4) | 16.5 (3.5 to 29.6) | 1.27 (1.07 to 1.51) | 1.22 (1.03 to 1.45) |
| IBD duration until index date, years |  |  |  |  |  |
| 0 | 291 (116.7) | 18 (54.6) | 62.0 (33.4 to 90.6) | 1.37 (0.85 to 2.22) | 1.37 (0.84 to 2.22) |
| 1-5 | 247 (83.2) | 175 (65.4) | 17.8 (3.6 to 32.0) | 1.08 (0.89 to 1.32) | 1.09 (0.89 to 1.34) |
| >5 | 330 (71.8) | 365 (75.6) | -3.8 (-14.8 to 7.2) | 1.04 (0.90 to 1.22) | 1.00 (0.86 to 1.17) |
| Educational attainment |  |  |  |  |  |
| 0-9 y | 339 (164.1) | 182 (125.8) | 38.3 (13.0 to 63.6) | 1.17 (0.97 to 1.41) | 1.20 (0.99 to 1.45) |
| 10-12 y | 313 (73.0) | 231 (67.4) | 5.6 (-6.2 to 17.5) | 1.19 (1.00 to 1.41) | 1.17 (0.98 to 1.40) |
| ≥13 y | 123 (46.5) | 128 (50.3) | -3.7 (-15.7 to 8.2) | 1.00 (0.77 to 1.29) | 0.94 (0.72 to 1.22) |
| Missing | 93 (87.6) | 17 (41.5) | 46.1 (19.6 to 72.7) | 1.40 (0.83 to 2.36) | 1.60 (0.94 to 2.73) |
| Number of healthcare visits |  |  |  |  |  |
| 0 | 325 (71.9) | 159 (60.1) | 11.7 (-0.5 to 23.9) | 1.08 (0.88 to 1.32) | 1.08 (0.89 to 1.32) |
| 1 | 176 (88.9) | 103 (60.3) | 28.6 (11.1 to 46.2) | 1.32 (1.02 to 1.70) | 1.27 (0.98 to 1.65) |
| 2-3 | 179 (99.7) | 121 (73.1) | 26.7 (7.1 to 46.2) | 1.37 (1.08 to 1.73) | 1.37 (1.07 to 1.74) |
| ≥4 | 188 (106.9) | 175 (96.1) | 10.8 (-10.1 to 31.7) | 1.11 (0.90 to 1.36) | 1.10 (0.89 to 1.35) |
| Hypertension |  |  |  |  |  |
| No | 658 (69.1) | 398 (54.7) | 14.4 (6.9 to 22.0) | 1.19 (1.04 to 1.35) | 1.17 (1.03 to 1.33) |
| Yes | 210 (391.3) | 160 (288.9) | 102.4 (33.1 to 171.7) | 1.13 (0.91 to 1.40) | 1.13 (0.91 to 1.40) |
| Dyslipidemia |  |  |  |  |  |
| No | 839 (84.1) | 536 (69.4) | 14.6 (6.5 to 22.8) | 1.16 (1.04 to 1.30) | 1.15 (1.02 to 1.29) |
| Yes | 29 (372.5) | 22 (197.1) | 175.4 (16.8 to 334.1) | 1.77 (1.00 to 3.12) | 1.69 (0.95 to 3.00) |
| Diabetes |  |  |  |  |  |
| No | 774 (79.8) | 489 (65.1) | 14.7 (6.6 to 22.7) | 1.17 (1.04 to 1.32) | 1.16 (1.03 to 1.30) |
| Yes | 94 (266.6) | 69 (219.1) | 47.5 (-27.2 to 122.2) | 1.27 (0.93 to 1.74) | 1.30 (0.95 to 1.78) |
| Obesity |  |  |  |  |  |
| No | 846 (85.2) | 541 (70.2) | 14.9 (6.7 to 23.2) | 1.17 (1.04 to 1.31) | 1.15 (1.03 to 1.29) |
| Yes | 22 (182.6) | 17 (134.0) | 48.7 (-50.8 to 148.1) | 1.47 (0.77 to 2.82) | 1.39 (0.72 to 2.69) |
| Chronic kidney diseases |  |  |  |  |  |
| No | 841 (84.3) | 543 (70.0) | 14.3 (6.1 to 22.5) | 1.16 (1.03 to 1.30) | 1.15 (1.02 to 1.29) |
| Yes | 27 (332.8) | 15 (208.4) | 124.4 (-39.5 to 288.4) | 1.68 (0.87 to 3.25) | 1.60 (0.82 to 3.11) |
| COPD |  |  |  |  |  |
| No | 810 (81.4) | 529 (68.3) | 13.2 (5.1 to 21.3) | 1.15 (1.03 to 1.29) | 1.14 (1.02 to 1.28) |
| Yes | 58 (530.4) | 29 (363.3) | 167.1 (-22.9 to 357.2) | 1.54 (0.97 to 2.45) | 1.57 (0.98 to 2.51) |
| CI: confidence interval; HR: hazard ratio; IR: incidence rate; MACE: major adverse cardiovascular events. | | | | | |
| ^a^ Adjusted for age at IBD diagnosis, sex, and calendar year at index date. | | | | | |
| ^b^ Further adjusted for county of residence, educational attainment, country of birth, number of health care visits, hypertension, diabetes, obesity, dyslipidemia, chronic kidney disease, and COPD. | | | | | |

| Table S11. Risk of 2-year MACE for histologic inflammation and histologic remission in patients with IBD, stratified by the phenotypes of the Montreal Classification | | | | | |
| --- | --- | --- | --- | --- | --- |
|  | No. of events (IR, per 10,000 Pys) | | IR difference (95%CI), per 10,000 Pys | HR (95%CIs) | |
|  | Histologic inflammation | Histologic remission |  | Model 1 ^a^ | Model 2 ^b^ |
| IBD |  |  |  |  |  |
| Primary sclerosing cholangitis | 9 (91.1) | 2 (21.4) | 69.6 (3.1 to 136.2) | 4.15 (0.88 to 19.62) | 7.22 (1.02 to 51.04) |
| Other extraintestinal manifestations | 31 (159.8) | 13 (72.4) | 87.4 (18.8 to 156.1) | 1.99 (1.02 to 3.85) | 2.11 (1.08 to 4.13) |
| CD |  |  |  |  |  |
| No location information | 77 (67.9) | 63 (77.5) | -9.6 (-34.0 to 14.9) | 1.03 (0.72 to 1.47) | 0.97 (0.68 to 1.39) |
| L1, L3/LX (Ileal, ileocolonic or location not defined) | 89 (84.0) | 48 (48.0) | 35.9 (13.8 to 58.0) | 1.56 (1.09 to 2.24) | 1.58 (1.10 to 2.28) |
| L2 (Colonic) | 31 (87.9) | 18 (47.1) | 40.7 (2.9 to 78.6) | 1.96 (1.08 to 3.56) | 1.96 (1.08 to 3.56) |
| Perianal | 8 (75.5) | 2 (23.9) | 51.6 (-10.3 to 113.5) | 2.20 (0.38 to 12.85) | NA |
| Primary sclerosing cholangitis | 0 (0.0) | 0 (0.0) | NA | NA | NA |
| Other extraintestinal manifestations | 7 (102.1) | 3 (41.0) | 61.1 (-27.6 to 149.8) | 3.28 (0.70 to 15.30) | NA |
| UC |  |  |  |  |  |
| No extent information ^c^ | 251 (82.9) | 181 (93.2) | -10.2 (-27.2 to 6.8) | 0.94 (0.77 to 1.16) | 0.96 (0.78 to 1.18) |
| E1/E2 (Proctitis, left-sided colitis) | 108 (87.9) | 64 (62.4) | 25.6 (3.0 to 48.1) | 1.18 (0.85 to 1.62) | 1.15 (0.83 to 1.60) |
| E3 (Extensive colitis) | 64 (72.7) | 46 (58.1) | 14.5 (-10.0 to 39.0) | 1.26 (0.85 to 1.87) | 1.24 (0.82 to 1.86) |
| EX (Extent not defined) | 188 (103.4) | 108 (73.0) | 30.4 (10.2 to 50.6) | 1.25 (0.98 to 1.60) | 1.22 (0.95 to 1.57) |
| Primary sclerosing cholangitis | 9 (106.9) | 2 (24.5) | 82.3 (4.7 to 160.0) | 7.46 (1.07 to 52.30) | NA |
| Other extraintestinal manifestations | 19 (173.9) | 9 (95.8) | 78.1 (-22.1 to 178.3) | 1.70 (0.73 to 3.97) | NA |
| CD: Crohn’s disease; CI: confidence interval; E: Extent; HR: hazard ratio; IR: incident rate; L: location; MACE: major adverse cardiovascular events; NA: not available; UC: ulcerative colitis. | | | | |  |
| ^a^ Adjusted for age at IBD diagnosis, sex, and calendar year at index date. | | | | | |
| ^b^ Further adjusted for county of residence, educational attainment, country of birth, number of health care visits, and Charlson comorbidity index. | | | | | |
| ^c^ No extent information refers to patients who were diagnosed with UC before the introduction of ICD-10 in 1997. | | | | | |

| Table S12. Risk of 5-year MACE for histologic inflammation and histologic remission in patients with IBD | | | | | |
| --- | --- | --- | --- | --- | --- |
| Outcomes | No. of events (IR, per 10,000 person-years) | | IR difference (95%CIs), per 10,000 person-years | HR (95%CIs) | |
|  | Histologic inflammation | Histologic remission |  | Model 1 ^a^ | Model 2 ^b^ |
| MACE | 879 (86.5) | 1170 (75.2) | 11.3 (4.1 to 18.4) | 1.21 (1.10 to 1.33) | 1.17 (1.07 to 1.28) |
| Ischemic heart disease | 532 (51.2) | 694 (43.6) | 7.6 (2.2 to 13.1) | 1.18 (1.05 to 1.33) | 1.15 (1.02 to 1.29) |
| Myocardial infarction | 336 (31.6) | 371 (22.6) | 9.0 (4.9 to 13.1) | 1.44 (1.23 to 1.68) | 1.41 (1.21 to 1.65) |
| Stroke | 321 (30.0) | 413 (25.1) | 4.9 (0.8 to 9.0) | 1.28 (1.09 to 1.49) | 1.21 (1.04 to 1.42) |
| Hemorrhagic stroke | 64 (5.9) | 91 (5.5) | 0.5 (-1.4 to 2.3) | 1.21 (0.87 to 1.69) | 1.14 (0.81 to 1.60) |
| Ischemic stroke | 251 (23.4) | 325 (19.7) | 3.7 (0.1 to 7.3) | 1.31 (1.10 to 1.56) | 1.26 (1.06 to 1.50) |
| Heart failure | 474 (44.3) | 506 (30.7) | 13.6 (8.8 to 18.4) | 1.45 (1.27 to 1.66) | 1.40 (1.22 to 1.59) |
| CI: confidence interval; HR: hazard ratio; IR: incidence rate; MACE: major adverse cardiovascular events. | | | | | |
| ^a^ Adjusted for age at IBD diagnosis, sex, and calendar year at index date. | | | | | |
| ^b^ Further adjusted for county of residence, educational attainment, country of birth, number of health care visits, hypertension, diabetes, obesity, dyslipidemia, chronic kidney disease, and COPD. | | | | | |

| Table S13. Risk of 2-year MACE for histologic inflammation and histologic remission in patients with IBD ^a^ | | | | | |
| --- | --- | --- | --- | --- | --- |
| Outcomes | No. of events (IR, per 10,000 person-years) | | IR difference (95%CIs), per 10,000 person-years | HR (95%CIs) | |
|  | Histologic inflammation | Histologic remission |  | Model 1 ^b^ | Model 2 ^c^ |
| MACE | 502 (94.7) | 558 (71.3) | 23.4 (13.2 to 33.6) | 1.31 (1.15 to 1.48) | 1.28 (1.13 to 1.45) |
| Ischemic heart disease | 295 (54.5) | 331 (41.4) | 13.1 (5.4 to 20.7) | 1.26 (1.07 to 1.48) | 1.22 (1.04 to 1.44) |
| Myocardial infarction | 195 (35.2) | 182 (22.2) | 13.0 (7.1 to 18.9) | 1.53 (1.24 to 1.89) | 1.49 (1.20 to 1.85) |
| Stroke | 180 (32.3) | 193 (23.5) | 8.9 (3.1 to 14.6) | 1.34 (1.08 to 1.66) | 1.28 (1.03 to 1.59) |
| Hemorrhagic stroke | 36 (6.4) | 39 (4.7) | 1.7 (-0.9 to 4.2) | 1.35 (0.84 to 2.16) | 1.24 (0.77 to 2.00) |
| Ischemic stroke | 147 (26.3) | 146 (17.7) | 8.6 (3.5 to 13.7) | 1.49 (1.18 to 1.90) | 1.45 (1.14 to 1.85) |
| Heart failure | 298 (53.4) | 238 (28.9) | 24.5 (17.5 to 31.6) | 1.71 (1.43 to 2.04) | 1.65 (1.37 to 1.98) |
| CI: confidence interval; HR: hazard ratio; IR: incidence rate; MACE: major adverse cardiovascular events. | | | | | |
| ^a^ A histologic inflammation period was considered present six months following the biopsy date. | | | | | |
| ^b^ Adjusted for age at IBD diagnosis, sex, and calendar year at index date. | | | | | |
| ^c^ Further adjusted for county of residence, educational attainment, country of birth, number of health care visits, hypertension, diabetes, obesity, dyslipidemia, chronic kidney disease, and COPD. | | | | | |

| Table S14. Risk of 2-year MACE for histologic inflammation and histologic remission during periods of clinically quiescent IBD | | | | | |
| --- | --- | --- | --- | --- | --- |
| Outcomes | No. of events (IR, per 10,000 person-years) | | IR difference (95%CIs), per 10,000 person-years | HR (95%CIs) | |
|  | Histologic inflammation | Histologic remission |  | Model 1 ^a^ | Model 2 ^b^ |
| MACE | 484 (72.1) | 396 (70.3) | 1.8 (-7.7 to 11.2) | 1.06 (0.94 to 1.19) | 1.05 (0.93 to 1.18) |
| Ischemic heart disease | 312 (45.6) | 251 (43.7) | 1.9 (-5.5 to 9.3) | 1.07 (0.92 to 1.24) | 1.07 (0.92 to 1.24) |
| Myocardial infarction | 189 (27.0) | 131 (22.2) | 4.8 (-0.6 to 10.2) | 1.25 (1.03 to 1.53) | 1.29 (1.06 to 1.58) |
| Stroke | 182 (25.8) | 132 (22.3) | 3.5 (-1.8 to 8.9) | 1.14 (0.94 to 1.39) | 1.11 (0.91 to 1.35) |
| Hemorrhagic stroke | 35 (4.9) | 32 (5.3) | -0.4 (-2.9 to 2.0) | 1.06 (0.69 to 1.64) | 1.01 (0.65 to 1.56) |
| Ischemic stroke | 132 (18.7) | 103 (17.4) | 1.3 (-3.3 to 5.9) | 1.05 (0.84 to 1.32) | 1.04 (0.83 to 1.31) |
| Heart failure | 210 (29.7) | 143 (24.1) | 5.6 (0.0 to 11.3) | 1.16 (0.97 to 1.39) | 1.19 (1.00 to 1.43) |
| CI: confidence interval; HR: hazard ratio; IR: incidence rate; MACE: major adverse cardiovascular events. | | | | | |
| ^a^ Adjusted for age at IBD diagnosis, sex, and calendar year at index date. | | | | | |
| ^b^ Further adjusted for county of residence, educational attainment, country of birth, number of health care visits, hypertension, diabetes, obesity, dyslipidemia, chronic kidney disease, and COPD. | | | | | |

| Table S15. Sensitivity analyses of the 2-year MACE risk for histologic inflammation and histologic remission in patients with IBD | | | | | | | |
| --- | --- | --- | --- | --- | --- | --- | --- |
| Outcomes | After excluding the first 3 months after IBD diagnosis | | |  | After excluding the first 1 year after IBD diagnosis | | |
|  | No. of events (IR, per 10,000 person-years) | | HR (95%CIs) ^a^ |  | No. of events (IR, per 10,000 person-years) | | HR (95%CIs) ^a^ |
|  | Histologic inflammation | Histologic remission |  |  | Histologic inflammation | Histologic remission |  |
| MACE | 735 (79.1) | 557 (71.4) | 1.08 (0.96 to 1.21) |  | 527 (74.7) | 540 (70.3) | 1.11 (0.98 to 1.26) |
| Ischemic heart disease | 441 (46.5) | 334 (41.9) | 1.05 (0.91 to 1.22) |  | 325 (45.1) | 323 (41.2) | 1.14 (0.97 to 1.33) |
| Myocardial infarction | 271 (27.9) | 183 (22.4) | 1.22 (1.00 to 1.49) |  | 196 (26.6) | 179 (22.2) | 1.28 (1.03 to 1.58) |
| Stroke | 276 (28.3) | 191 (23.3) | 1.15 (0.95 to 1.40) |  | 192 (25.9) | 194 (24.0) | 1.11 (0.90 to 1.37) |
| Hemorrhagic stroke | 56 (5.7) | 39 (4.7) | 1.17 (0.76 to 1.78) |  | 38 (5.1) | 38 (4.6) | 1.13 (0.71 to 1.80) |
| Ischemic stroke | 214 (21.8) | 145 (17.6) | 1.23 (0.99 to 1.54) |  | 149 (20.0) | 149 (18.4) | 1.18 (0.93 to 1.49) |
| Heart failure | 388 (39.7) | 236 (28.7) | 1.27 (1.07 to 1.50) |  | 252 (33.9) | 235 (29.0) | 1.20 (1.00 to 1.44) |
| CI: confidence interval; HR: hazard ratio; IR: incidence rate. | | | | | | | |
| ^a^ Adjusted for age at IBD diagnosis, sex, calendar year at index date, county of residence, educational attainment, country of birth, number of health care visits, hypertension, diabetes, obesity, dyslipidemia, chronic kidney disease, and COPD. | | | | | | | |

| Table S16. Baseline characteristics of IBD patients in the cohort of clinically active and quiescent IBD in 2006–2020 | | | | |
| --- | --- | --- | --- | --- |
|  | Patients with IBD | Subtypes of IBD | | |
|  |  | CD | UC | IBD-U |
| No. of patients | 91800 | 30729 | 54015 | 7056 |
| Age at IBD diagnosis, years |  |  |  |  |
| Mean ± SD | 39.2 ± 18.3 | 36.9 ± 18.0 | 40.3 ± 18.0 | 40.8 ± 20.6 |
| Median (IQR) | 36.1 (24.6-52.5) | 33.2 (22.3-50.0) | 37.5 (26.1-53.3) | 36.9 (23.3-57.5) |
| <18 | 9916 (10.8) | 4295 (14.0) | 4654 (8.6) | 967 (13.7) |
| 18-<40 | 42192 (46.0) | 14565 (47.4) | 24787 (45.9) | 2840 (40.3) |
| 40-<60 | 25027 (27.3) | 7720 (25.1) | 15618 (28.9) | 1689 (23.9) |
| ≥60 | 14665 (16.0) | 4149 (13.5) | 8956 (16.6) | 1560 (22.1) |
| Female | 46116 (50.2) | 16068 (52.3) | 26347 (48.8) | 3701 (52.5) |
| Born in Nordic country | 82787 (90.2) | 27357 (89.0) | 49066 (90.8) | 6364 (90.2) |
| Calendar period at IBD diagnosis |  |  |  |  |
| 1969-1989 | 8595 (9.4) | 3727 (12.1) | 4126 (7.6) | 742 (10.5) |
| 1990-2001 | 16290 (17.8) | 5523 (18.0) | 10319 (19.1) | 448 (6.4) |
| 2002-2009 | 24928 (27.2) | 7948 (25.9) | 15917 (29.5) | 1063 (15.1) |
| 2010-2020 | 41987 (45.7) | 13531 (44.0) | 23653 (43.8) | 4803 (68.1) |
| Educational attainment |  |  |  |  |
| 0-9 y | 17963 (19.6) | 6437 (21.0) | 10084 (18.7) | 1442 (20.4) |
| 10-12 y | 39119 (42.6) | 13096 (42.6) | 23243 (43.0) | 2780 (39.4) |
| ≥13 y | 26001 (28.3) | 7864 (25.6) | 16422 (30.4) | 1715 (24.3) |
| Missing | 8717 (9.5) | 3332 (10.8) | 4266 (7.9) | 1119 (15.9) |
| Montreal Classification CD at IBD diagnosis |  |  |  |  |
| No location information | - | 6147 (20.0) | - | - |
| L1, L3/LX (Ileal, ileocolonic or location not defined) | - | 20080 (65.4) | - | - |
| L2 (Colonic) | - | 4502 (14.7) | - | - |
| Perianal | - | 1676 (5.5) | - | - |
| Montreal Classification UC at IBD diagnosis |  |  |  |  |
| No extent information ^a^ | - | - | 8113 (15.0) | - |
| E1/E2 (Proctitis, left-sided colitis) | - | - | 19342 (35.8) | - |
| E3 (Extensive colitis) | - | - | 8640 (16.0) | - |
| EX (Extent not defined) | - | - | 17920 (33.2) | - |
| Extraintestinal manifestations at IBD diagnosis |  |  |  |  |
| Primary sclerosing cholangitis | 743 (0.8) | 135 (0.4) | 553 (1.0) | 55 (0.8) |
| Other extraintestinal manifestations | 4960 (5.4) | 2016 (6.6) | 2384 (4.4) | 560 (7.9) |
| CD: Crohn’s disease; IBD(-U): inflammatory bowel disease (unclassified); IQR: interquartile range; SD: standard deviation; UC: ulcerative colitis. | | | | |
| ^a^ No extent information refers to patients who were diagnosed with UC before the introduction of ICD-10 in 1997. | | | | |

| Table S17. Risk of 2-year MACE for clinically active and quiescent IBD in patients with IBD | | | | | |
| --- | --- | --- | --- | --- | --- |
| Outcomes | No. of events (IR, per 10,000 person-years) | | IR difference (95%CIs), per 10,000 person-years | HR (95%CIs) | |
|  | Active IBD | Quiescent IBD |  | Model 1 ^a^ | Model 2 ^b^ |
| MACE | 2599 (131.4) | 2274 (93.7) | 37.7 (31.3 to 44.1) | 1.56 (1.48 to 1.65) | 1.54 (1.46 to 1.63) |
| Ischemic heart disease | 1323 (64.2) | 1363 (53.7) | 10.5 (6.0 to 14.9) | 1.33 (1.24 to 1.44) | 1.32 (1.22 to 1.43) |
| Myocardial infarction | 883 (41.2) | 750 (28.3) | 12.9 (9.5 to 16.3) | 1.63 (1.48 to 1.79) | 1.62 (1.47 to 1.79) |
| Stroke | 983 (45.7) | 921 (34.6) | 11.1 (7.5 to 14.7) | 1.47 (1.34 to 1.60) | 1.44 (1.31 to 1.57) |
| Hemorrhagic stroke | 215 (9.8) | 204 (7.5) | 2.3 (0.6 to 4.0) | 1.43 (1.18 to 1.74) | 1.40 (1.15 to 1.70) |
| Ischemic stroke | 827 (38.2) | 771 (28.8) | 9.4 (6.1 to 12.7) | 1.47 (1.34 to 1.63) | 1.45 (1.31 to 1.60) |
| Heart failure | 1928 (90.2) | 1241 (46.8) | 43.4 (38.6 to 48.2) | 2.17 (2.02 to 2.33) | 2.16 (2.01 to 2.32) |
| CI: confidence interval; HR: hazard ratio; IR: incidence rate; MACE: major adverse cardiovascular events. | | | | | |
| ^a^ Adjusted for age at IBD diagnosis, sex, and calendar year at index date. | | | | | |
| ^b^ Further adjusted for county of residence, educational attainment, country of birth, number of health care visits, hypertension, diabetes, obesity, dyslipidemia, chronic kidney disease, and COPD. | | | | | |

| Table S18. Risk of 2-year MACE for clinically active and quiescent IBD in patients with CD, UC, and IBD-U | | | | | |
| --- | --- | --- | --- | --- | --- |
| Outcomes | No. of events (IR, per 10,000 person-years) | | IR difference (95%CIs), per 10,000 person-years | HR (95%CIs) | |
|  | Active IBD | Quiescent IBD |  | Model 1 ^a^ | Model 2 ^b^ |
|  | Patients with CD | | | | |
| MACE | 929 (116.9) | 679 (88.6) | 28.3 (18.3 to 38.4) | 1.51 (1.37 to 1.66) | 1.50 (1.36 to 1.65) |
| Ischemic heart disease | 461 (55.7) | 372 (46.3) | 9.4 (2.5 to 16.3) | 1.37 (1.20 to 1.58) | 1.38 (1.20 to 1.58) |
| Myocardial infarction | 283 (33.1) | 199 (23.8) | 9.3 (4.2 to 14.3) | 1.60 (1.33 to 1.92) | 1.60 (1.33 to 1.91) |
| Stroke | 337 (39.5) | 272 (32.7) | 6.8 (1.0 to 12.5) | 1.38 (1.17 to 1.62) | 1.36 (1.15 to 1.59) |
| Hemorrhagic stroke | 81 (9.3) | 55 (6.4) | 2.8 (0.2 to 5.5) | 1.61 (1.14 to 2.27) | 1.56 (1.11 to 2.21) |
| Ischemic stroke | 272 (31.7) | 236 (28.2) | 3.5 (-1.7 to 8.7) | 1.29 (1.09 to 1.54) | 1.28 (1.07 to 1.52) |
| Heart failure | 637 (75.0) | 356 (42.9) | 32.1 (24.8 to 39.4) | 2.05 (1.80 to 2.33) | 2.04 (1.79 to 2.32) |
|  | Patients with UC | | | | |
| MACE | 1451 (137.0) | 1436 (95.4) | 41.6 (33.0 to 50.2) | 1.55 (1.44 to 1.67) | 1.53 (1.42 to 1.65) |
| Ischemic heart disease | 761 (68.9) | 874 (55.6) | 13.3 (7.1 to 19.4) | 1.34 (1.21 to 1.47) | 1.33 (1.20 to 1.46) |
| Myocardial infarction | 526 (45.7) | 494 (30.1) | 15.7 (10.9 to 20.4) | 1.64 (1.45 to 1.86) | 1.64 (1.45 to 1.86) |
| Stroke | 555 (47.9) | 582 (35.2) | 12.7 (7.8 to 17.6) | 1.46 (1.30 to 1.64) | 1.43 (1.28 to 1.61) |
| Hemorrhagic stroke | 124 (10.4) | 135 (7.9) | 2.5 (0.2 to 4.8) | 1.41 (1.10 to 1.80) | 1.39 (1.09 to 1.78) |
| Ischemic stroke | 474 (40.6) | 475 (28.5) | 12.1 (7.6 to 16.6) | 1.52 (1.34 to 1.73) | 1.50 (1.32 to 1.70) |
| Heart failure | 1120 (97.1) | 793 (48.1) | 49.1 (42.5 to 55.7) | 2.18 (1.99 to 2.39) | 2.18 (1.99 to 2.39) |
|  | Patients with IBD-U | | | | |
| MACE | 219 (176.3) | 159 (102.1) | 74.3 (46.1 to 102.5) | 1.99 (1.62 to 2.44) | 1.91 (1.56 to 2.35) |
| Ischemic heart disease | 101 (77.6) | 117 (71.1) | 6.4 (-13.5 to 26.3) | 1.23 (0.94 to 1.61) | 1.18 (0.90 to 1.55) |
| Myocardial infarction | 74 (54.1) | 57 (33.0) | 21.1 (6.1 to 36.1) | 1.83 (1.29 to 2.58) | 1.74 (1.22 to 2.46) |
| Stroke | 91 (66.4) | 67 (38.7) | 27.6 (11.1 to 44.1) | 1.97 (1.43 to 2.70) | 1.88 (1.37 to 2.59) |
| Hemorrhagic stroke | 10 (7.1) | 14 (7.8) | -0.7 (-6.7 to 5.3) | 0.99 (0.44 to 2.24) | 0.92 (0.41 to 2.10) |
| Ischemic stroke | 81 (58.6) | 60 (34.4) | 24.3 (8.8 to 39.7) | 1.96 (1.40 to 2.75) | 1.90 (1.35 to 2.66) |
| Heart failure | 171 (125.3) | 92 (53.2) | 72.1 (50.4 to 93.8) | 2.70 (2.10 to 3.49) | 2.65 (2.06 to 3.43) |
| CI: confidence interval; HR: hazard ratio; IR: incidence rate; MACE: major adverse cardiovascular events; NA: not available. | | | | | |
| ^a^ Adjusted for age at IBD diagnosis, sex, and calendar year at index date. | | | | | |
| ^b^ Further adjusted for county of residence, educational attainment, country of birth, number of health care visits, hypertension, diabetes, obesity, dyslipidemia, chronic kidney disease, and COPD. | | | | | |

| Table S19. Subgroup analyses for risk of 2-year MACE for clinically active and quiescent IBD in patients with IBD | | | | | |
| --- | --- | --- | --- | --- | --- |
| Outcomes | No. of events (IR, per 10,000 person-years) | | IR difference (95%CIs), per 10,000 person-years | HR (95%CIs) | |
|  | Active IBD | Quiescent IBD |  | Model 1 ^a^ | Model 2 ^b^ |
| Sex |  | | | | |
| Male | 1341 (137.6) | 1248 (110.2) | 27.5 (17.9 to 37.0) | 1.45 (1.35 to 1.57) | 1.45 (1.34 to 1.57) |
| Female | 1258 (125.4) | 1026 (79.3) | 46.1 (37.6 to 54.5) | 1.70 (1.56 to 1.84) | 1.65 (1.52 to 1.79) |
| Age at IBD diagnosis, years |  |  |  |  |  |
| <18 | 44 (15.4) | 28 (11.0) | 4.3 (-1.8 to 10.4) | 1.72 (1.07 to 2.78) | 1.62 (1.00 to 2.63) |
| 18-<40 | 484 (50.1) | 419 (35.7) | 14.3 (8.7 to 19.9) | 1.54 (1.35 to 1.75) | 1.48 (1.30 to 1.69) |
| 40-<60 | 857 (170.4) | 830 (120.8) | 49.6 (35.6 to 63.7) | 1.40 (1.27 to 1.54) | 1.40 (1.27 to 1.54) |
| ≥60 | 1214 (548.0) | 997 (318.1) | 229.9 (193.3 to 266.5) | 1.70 (1.57 to 1.85) | 1.68 (1.54 to 1.82) |
| Age at index date, years |  |  |  |  |  |
| <18 | 8 (7.4) | 3 (4.3) | 3.1 (-3.9 to 10.2) | 1.66 (0.44 to 6.26) | 1.94 (0.50 to 7.45) |
| 18-<40 | 76 (10.7) | 37 (4.5) | 6.2 (3.4 to 9.0) | 2.42 (1.63 to 3.59) | 2.38 (1.60 to 3.53) |
| 40-<60 | 463 (71.2) | 465 (55.2) | 16.0 (7.8 to 24.2) | 1.30 (1.15 to 1.48) | 1.27 (1.12 to 1.45) |
| ≥60 | 2052 (402.3) | 1769 (255.0) | 147.3 (126.2 to 168.4) | 1.61 (1.51 to 1.72) | 1.59 (1.49 to 1.69) |
| Calendar period at IBD diagnosis |  |  |  |  |  |
| 1969-1989 | 498 (234.4) | 454 (161.9) | 72.5 (47.1 to 97.9) | 1.58 (1.39 to 1.79) | 1.51 (1.33 to 1.72) |
| 1990-2001 | 632 (138.3) | 591 (110.3) | 27.9 (14.0 to 41.9) | 1.35 (1.21 to 1.51) | 1.36 (1.22 to 1.52) |
| 2002-2009 | 830 (123.3) | 687 (82.8) | 40.6 (30.1 to 51.0) | 1.64 (1.48 to 1.81) | 1.63 (1.47 to 1.80) |
| 2010-2017 | 639 (100.5) | 542 (69.4) | 31.1 (21.4 to 40.9) | 1.70 (1.52 to 1.91) | 1.65 (1.47 to 1.85) |
| Calendar period at index date |  |  |  |  |  |
| 2002-2009 | 561 (132.3) | 465 (85.7) | 46.6 (33.1 to 60.0) | 1.71 (1.52 to 1.94) | 1.68 (1.48 to 1.90) |
| 2010-2021 | 2038 (131.1) | 1809 (96.0) | 35.2 (27.9 to 42.4) | 1.53 (1.44 to 1.63) | 1.51 (1.42 to 1.61) |
| IBD duration until index date, years |  |  |  |  |  |
| 0 | 326 (119.8) | 209 (65.0) | 54.8 (39.1 to 70.5) | 2.17 (1.82 to 2.58) | 1.94 (1.63 to 2.32) |
| 1-5 | 531 (100.8) | 535 (74.9) | 25.9 (15.2 to 36.5) | 1.56 (1.39 to 1.76) | 1.57 (1.39 to 1.77) |
| >5 | 1742 (147.8) | 1530 (110.0) | 37.8 (28.9 to 46.6) | 1.48 (1.39 to 1.59) | 1.47 (1.38 to 1.58) |
| Educational attainment |  |  |  |  |  |
| 0-9 y | 887 (238.5) | 781 (174.8) | 63.7 (43.8 to 83.6) | 1.54 (1.40 to 1.69) | 1.54 (1.40 to 1.70) |
| 10-12 y | 1135 (125.9) | 964 (85.5) | 40.3 (31.3 to 49.4) | 1.62 (1.49 to 1.77) | 1.61 (1.48 to 1.76) |
| ≥13 y | 459 (75.5) | 463 (59.1) | 16.4 (7.6 to 25.2) | 1.39 (1.22 to 1.58) | 1.38 (1.21 to 1.57) |
| Missing | 118 (122.3) | 66 (94.7) | 27.5 (-4.2 to 59.3) | 1.59 (1.17 to 2.17) | 1.54 (1.13 to 2.09) |
| Number of healthcare visits |  |  |  |  |  |
| 0 | 470 (104.4) | 468 (74.9) | 29.4 (17.8 to 41.1) | 1.68 (1.47 to 1.91) | 1.64 (1.44 to 1.86) |
| 1 | 396 (104.3) | 365 (77.5) | 26.8 (13.8 to 39.8) | 1.53 (1.32 to 1.76) | 1.54 (1.34 to 1.78) |
| 2-3 | 601 (125.4) | 524 (91.0) | 34.5 (21.8 to 47.2) | 1.52 (1.35 to 1.70) | 1.52 (1.35 to 1.71) |
| ≥4 | 1132 (169.2) | 917 (121.4) | 47.8 (35.2 to 60.4) | 1.51 (1.38 to 1.65) | 1.50 (1.38 to 1.64) |
| Hypertension |  |  |  |  |  |
| No | 1533 (87.6) | 1288 (60.9) | 26.8 (21.3 to 32.3) | 1.58 (1.47 to 1.71) | 1.55 (1.44 to 1.67) |
| Yes | 1066 (467.0) | 986 (317.5) | 149.4 (115.1 to 183.8) | 1.55 (1.43 to 1.70) | 1.52 (1.39 to 1.66) |
| Dyslipidemia |  |  |  |  |  |
| No | 2442 (125.7) | 2111 (89.0) | 36.7 (30.4 to 43.0) | 1.57 (1.48 to 1.66) | 1.54 (1.45 to 1.63) |
| Yes | 157 (438.4) | 163 (290.4) | 148.1 (66.3 to 229.9) | 1.60 (1.28 to 1.99) | 1.54 (1.23 to 1.92) |
| Diabetes |  |  |  |  |  |
| No | 2185 (116.4) | 1893 (82.6) | 33.7 (27.6 to 39.9) | 1.57 (1.48 to 1.67) | 1.54 (1.45 to 1.64) |
| Yes | 414 (412.0) | 381 (280.0) | 132.0 (83.3 to 180.6) | 1.56 (1.36 to 1.80) | 1.54 (1.34 to 1.78) |
| Obesity |  |  |  |  |  |
| No | 2472 (128.9) | 2154 (91.8) | 37.1 (30.7 to 43.5) | 1.57 (1.48 to 1.66) | 1.54 (1.45 to 1.63) |
| Yes | 127 (209.6) | 120 (148.8) | 60.8 (15.7 to 105.9) | 1.58 (1.23 to 2.04) | 1.55 (1.20 to 1.99) |
| Chronic kidney diseases |  |  |  |  |  |
| No | 2417 (124.8) | 2129 (89.1) | 35.6 (29.4 to 41.9) | 1.56 (1.47 to 1.65) | 1.55 (1.46 to 1.64) |
| Yes | 182 (446.4) | 145 (377.1) | 69.3 (-20.0 to 158.6) | 1.45 (1.16 to 1.81) | 1.42 (1.14 to 1.78) |
| COPD |  |  |  |  |  |
| No | 2234 (116.1) | 2029 (86.0) | 30.2 (24.1 to 36.3) | 1.51 (1.43 to 1.61) | 1.50 (1.41 to 1.59) |
| Yes | 365 (673.5) | 245 (367.3) | 306.2 (223.2 to 389.2) | 1.87 (1.59 to 2.20) | 1.86 (1.58 to 2.18) |
| CI: confidence interval; HR: hazard ratio; IR: incidence rate; MACE: major adverse cardiovascular events. | | | | | |
| ^a^ Adjusted for age at IBD diagnosis, sex, and calendar year at index date. | | | | | |
| ^b^ Further adjusted for county of residence, educational attainment, country of birth, number of health care visits, hypertension, diabetes, obesity, dyslipidemia, chronic kidney disease, and COPD. | | | | | |

| Table S20. Risk of 2-year MACE for clinically active and quiescent IBD in patients with IBD, stratified by the phenotypes of the Montreal Classification | | | | | |
| --- | --- | --- | --- | --- | --- |
|  | No. of events (IR, per 10,000 Pys) | | IR difference (95%CI), per 10,000 Pys | HR (95%CIs) | |
|  | Active IBD | Quiescent IBD |  | Model 1 ^a^ | Model 2 ^b^ |
| IBD |  |  |  |  |  |
| Primary sclerosing cholangitis | 22 (117.1) | 17 (97.7) | 19.4 (-48.0 to 86.9) | 1.35 (0.70 to 2.60) | 1.39 (0.72 to 2.68) |
| Other extraintestinal manifestations | 143 (142.1) | 104 (101.1) | 41.0 (10.7 to 71.4) | 1.52 (1.18 to 1.96) | 1.51 (1.17 to 1.95) |
| CD |  |  |  |  |  |
| No location information | 368 (196.6) | 263 (133.4) | 63.1 (37.4 to 88.9) | 1.64 (1.40 to 1.92) | 1.61 (1.38 to 1.89) |
| L1, L3/LX (Ileal, ileocolonic or location not defined) | 465 (95.0) | 332 (72.4) | 22.5 (10.9 to 34.2) | 1.47 (1.27 to 1.69) | 1.46 (1.27 to 1.69) |
| L2 (Colonic) | 96 (81.5) | 84 (75.7) | 5.8 (-17.2 to 28.8) | 1.31 (0.97 to 1.76) | 1.33 (0.99 to 1.78) |
| Perianal | 23 (50.3) | 20 (57.1) | -6.9 (-39.3 to 25.5) | 1.14 (0.61 to 2.14) | 1.06 (0.56 to 2.00) |
| Primary sclerosing cholangitis | 5 (149.8) | 2 (68.3) | 81.5 (-80.3 to 243.3) | 3.19 (0.34 to 29.72) | 2.91 (0.32 to 26.80) |
| Other extraintestinal manifestations | 48 (100.9) | 36 (93.4) | 7.4 (-34.3 to 49.2) | 1.21 (0.79 to 1.88) | 1.21 (0.78 to 1.86) |
| UC |  |  |  |  |  |
| No extent information ^c^ | 362 (200.1) | 377 (139.9) | 60.2 (35.3 to 85.2) | 1.52 (1.31 to 1.75) | 1.49 (1.29 to 1.72) |
| E1/E2 (Proctitis, left-sided colitis) | 354 (124.9) | 394 (76.5) | 48.3 (33.3 to 63.4) | 1.58 (1.37 to 1.83) | 1.57 (1.36 to 1.81) |
| E3 (Extensive colitis) | 215 (108.6) | 156 (69.1) | 39.5 (21.4 to 57.7) | 1.80 (1.46 to 2.21) | 1.79 (1.46 to 2.21) |
| EX (Extent not defined) | 520 (131.0) | 509 (102.9) | 28.1 (13.8 to 42.5) | 1.45 (1.29 to 1.64) | 1.44 (1.28 to 1.63) |
| Primary sclerosing cholangitis | 16 (111.4) | 15 (110.4) | 1.0 (-77.2 to 79.1) | 1.14 (0.55 to 2.36) | 1.15 (0.55 to 2.40) |
| Other extraintestinal manifestations | 70 (161.3) | 54 (99.2) | 62.1 (16.0 to 108.2) | 1.72 (1.20 to 2.46) | 1.72 (1.20 to 2.47) |
| CD: Crohn’s disease; CI: confidence interval; E: Extent; HR: hazard ratio; IR: incident rate; L: location; NA: not available; UC: ulcerative colitis. | | | | |  |
| ^a^ Adjusted for age at IBD diagnosis, sex, and calendar year at index date. | | | | | |
| ^b^ Further adjusted for county of residence, educational attainment, country of birth, number of health care visits, and Charlson comorbidity index. | | | | | |
| ^c^ No extent information refers to patients who were diagnosed with UC before the introduction of ICD-10 in 1997. | | | | | |

| Table S21. Risk of 5-year MACE for clinically active and quiescent IBD in patients with IBD | | | | | |
| --- | --- | --- | --- | --- | --- |
| Outcomes | No. of events (IR, per 10,000 person-years) | | IR difference (95%CIs), per 10,000 person-years | HR (95%CIs) | |
|  | Active IBD | Quiescent IBD |  | Model 1 ^a^ | Model 2 ^b^ |
| MACE | 3072 (122.6) | 3455 (88.9) | 33.7 (28.4 to 38.9) | 1.63 (1.55 to 1.71) | 1.57 (1.50 to 1.65) |
| Ischemic heart disease | 1577 (60.4) | 2059 (50.8) | 9.7 (6.0 to 13.4) | 1.40 (1.31 to 1.49) | 1.35 (1.27 to 1.44) |
| Myocardial infarction | 1055 (39.0) | 1138 (26.9) | 12.1 (9.3 to 14.9) | 1.70 (1.56 to 1.85) | 1.66 (1.52 to 1.80) |
| Stroke | 1164 (42.9) | 1398 (33.0) | 9.9 (6.9 to 12.9) | 1.51 (1.40 to 1.64) | 1.45 (1.34 to 1.57) |
| Hemorrhagic stroke | 252 (9.1) | 302 (6.9) | 2.1 (0.8 to 3.5) | 1.49 (1.26 to 1.76) | 1.43 (1.21 to 1.69) |
| Ischemic stroke | 975 (35.7) | 1167 (27.3) | 8.4 (5.6 to 11.1) | 1.52 (1.40 to 1.66) | 1.47 (1.34 to 1.60) |
| Heart failure | 2237 (82.9) | 1806 (42.6) | 40.3 (36.3 to 44.2) | 2.33 (2.19 to 2.48) | 2.25 (2.12 to 2.40) |
| CI: confidence interval; HR: hazard ratio; IR: incidence rate; MACE: major adverse cardiovascular events. | | | | | |
| ^a^ Adjusted for age at IBD diagnosis, sex, and calendar year at index date. | | | | | |
| ^b^ Further adjusted for county of residence, educational attainment, country of birth, number of health care visits, hypertension, diabetes, obesity, dyslipidemia, chronic kidney disease, and COPD. | | | | | |

| Table S22. Risk of 2-year MACE for clinically active and quiescent IBD in patients with IBD ^a^ | | | | | |
| --- | --- | --- | --- | --- | --- |
| Outcomes | No. of events (IR, per 10,000 person-years) | | IR difference (95%CIs), per 10,000 person-years | HR (95%CIs) | |
|  | Active IBD | Quiescent IBD |  | Model 1 ^b^ | Model 2 ^c^ |
| MACE | 2492 (134.5) | 1849 (88.4) | 46.0 (39.4 to 52.7) | 1.67 (1.58 to 1.78) | 1.65 (1.56 to 1.76) |
| Ischemic heart disease | 1278 (66.0) | 1126 (51.5) | 14.4 (9.7 to 19.2) | 1.40 (1.29 to 1.51) | 1.38 (1.28 to 1.50) |
| Myocardial infarction | 808 (40.0) | 638 (28.0) | 12.1 (8.5 to 15.6) | 1.55 (1.40 to 1.72) | 1.54 (1.39 to 1.71) |
| Stroke | 942 (46.6) | 758 (33.2) | 13.4 (9.6 to 17.2) | 1.52 (1.38 to 1.67) | 1.49 (1.36 to 1.64) |
| Hemorrhagic stroke | 208 (10.0) | 164 (7.0) | 3.0 (1.3 to 4.8) | 1.55 (1.26 to 1.90) | 1.51 (1.23 to 1.86) |
| Ischemic stroke | 790 (38.8) | 634 (27.5) | 11.3 (7.8 to 14.7) | 1.52 (1.37 to 1.69) | 1.50 (1.35 to 1.66) |
| Heart failure | 1845 (91.7) | 975 (42.7) | 49.0 (44.0 to 54.0) | 2.36 (2.18 to 2.55) | 2.35 (2.17 to 2.54) |
| CI: confidence interval; HR: hazard ratio; IR: incidence rate; MACE: major adverse cardiovascular events. | | | | | |
| ^a^ A clinical active period was considered present six months and would extend for an additional six months if another proxy occurred within six months of the previous one. | | | | | |
| ^b^ Adjusted for age at IBD diagnosis, sex, and calendar year at index date. | | | | | |
| ^c^ Further adjusted for county of residence, educational attainment, country of birth, number of health care visits, hypertension, diabetes, obesity, dyslipidemia, chronic kidney disease, and COPD. | | | | | |

Reference

1. Ludvigsson JF, Andersson E, Ekbom A, et al. External review and validation of the Swedish national inpatient register. *BMC Public Health*. Jun 9 2011;11:450. doi:10.1186/1471-2458-11-450

2. Wettermark B, Hammar N, Fored CM, et al. The new Swedish Prescribed Drug Register--opportunities for pharmacoepidemiological research and experience from the first six months. *Pharmacoepidemiol Drug Saf*. Jul 2007;16(7):726-35. doi:10.1002/pds.1294

3. Ludvigsson JF, Almqvist C, Bonamy AK, et al. Registers of the Swedish total population and their use in medical research. *Eur J Epidemiol*. Feb 2016;31(2):125-36. doi:10.1007/s10654-016-0117-y

4. Brooke HL, Talback M, Hornblad J, et al. The Swedish cause of death register. *Eur J Epidemiol*. Sep 2017;32(9):765-773. doi:10.1007/s10654-017-0316-1

5. Ludvigsson JF, Lashkariani M. Cohort profile: ESPRESSO (Epidemiology Strengthened by histoPathology Reports in Sweden). *Clin Epidemiol*. 2019;11:101-114. doi:10.2147/CLEP.S191914

6. Ludvigsson JF, Andersson M, Bengtsson J, et al. Swedish Inflammatory Bowel Disease Register (SWIBREG) - a nationwide quality register. *Scand J Gastroenterol*. Sep 2019;54(9):1089-1101. doi:10.1080/00365521.2019.1660799

7. Ludvigsson JF, Svedberg P, Olén O, Bruze G, Neovius M. The longitudinal integrated database for health insurance and labour market studies (LISA) and its use in medical research. *European Journal of Epidemiology*. 2019-04-01 2019;34(4):423-437. doi:10.1007/s10654-019-00511-8

8. Olén O, Erichsen R, Sachs MC, et al. Colorectal cancer in ulcerative colitis: a Scandinavian population-based cohort study. *The Lancet*. 2020-01-01 2020;395(10218):123-131. doi:10.1016/s0140-6736(19)32545-0
